# Reduction of cardiovascular risk factors by the diet – Evaluation of the MoKaRi concept by a parallel-designed randomized study

**DOI:** 10.1186/s12944-025-02500-1

**Published:** 2025-03-08

**Authors:** Christine Dawczynski, Timo Drobner, Thomas Weidauer, Peter Schlattmann, Michael Kiehntopf, Daniela Weber, Tilman Grune, Winfried März, Marcus E. Kleber, Stefan Lorkowski

**Affiliations:** 1 Junior Research Group Nutritional Concepts, Institute of Nutritional Sciences, Friedrich Schiller University Jena, Jena, 07743 Germany; 2 Competence Cluster for Nutrition and Cardiovascular Health (Nutricard) , Halle-Jena-Leipzig, Jena, 07743 Germany; 3 Department of Medical Statistics, Informatics and Data Science, University Hospital Jena, Jena, 07743 Germany; 4 Institute of Clinical Chemistry and Laboratory Diagnostics, University Hospital Jena, Jena, 07747 Germany; 5 Department of Molecular Toxicology, German Institute of Human Nutrition Potsdam-Rehbruecke (DIfE), Nuthetal, 14558 Germany; 6 German Center for Cardiovascular Research (DZHK), Partner Site Berlin, Berlin, Germany; 7 Medical Clinic V, Medical Faculty Mannheim, University of Heidelberg, Mannheim, 68167 Germany; 8 SYNLAB Academy, SYNLAB Holding Deutschland GmbH, Mannheim, 68161 Germany; 9 University of Heidelberg, Heidelberg, 69120 Germany; 10 Department of Nutritional Biochemistry and Physiology, Institute of Nutritional Sciences, Friedrich Schiller University Jena, Jena, 07743 Germany

**Keywords:** Nutritional concepts, Cardiovascular risk, Fatty acids, Dietary fibers

## Abstract

**Background and aim:**

The MoKaRi study aims to evaluate the impact of two nutritional concepts on cardiometabolic risk factors.

**Methods:**

For our 20-week intervention study, 65 participants with moderate elevated low-density lipoprotein cholesterol (LDL-C; ≥ 3 mmol/l) and without lipid-lowering therapy were recruited. The intervention to improve nutritional behavior was based on individualized menu plans which were characterized by defined energy and nutrient intake. To improve compliance, individual nutritional counselling sessions were held every two weeks. In addition to motivation, cooking skills were strengthened and nutritional knowledge was imparted. Follow-up visits were carried out after 10 and 20 weeks.

**Results:**

The MoKaRi diet lowered the concentrations of total cholesterol (menu plan group (MP): -15%; menu plan plus fish oil group (MP-FO): -11%), LDL-C (MP: -14%; MP-FO: -16%) and non-high-density lipoprotein cholesterol (MP: -16%; MP-FO: -13%) (*p* < 0.001). Body weight (MP: -5%; MP-FO: -8%; *p* < 0.05), waist circumference (MP: -6%; MP-FO: -9%) as well as diastolic blood pressure (MP: -8%; MP-FO: -8%), apolipoprotein A1 (MP: -15%; MP-FO: -20%), apolipoprotein B (MP: -15%; MP-FO: -6%) and glycated hemoglobin A_1c_ (HbA1c) (MP: -1.8%; MP-FO: -3.6%) were also reduced in both groups after 20 weeks (*p* < 0.05). In both intervention groups, a maximum reduction in LDL-c of approx. 26% was achieved within the 20 weeks of intervention. Individual participants achieved a reduction of 45–49%. The supplementation of fish oil on top of the menu plans resulted in more substantial effects on body weight (MP: -5% vs. MP-FO: -8%), body fat (MP: -11% vs. MP-FO: -20%), triglycerides (MP: -14% vs. MP-FO: -28%), high-sensitivity C-reactive protein (MP: -19% vs. MP-FO: -43%) and HbA1c (MP: -1.8% vs. MP-FO:—3.6%; *p* < 0.05).

**Conclusions:**

The MoKaRi diet resulted in a significant reduction of cardiometabolic risk factors. Our data highlights the additional benefit of the combination between menu plans and fish oil supplementation, which resulted in more substantial effects on body weight, BMI, TG, HbA1c and hs-CRP.

**ClinicalTrials.gov Identifier:**

NCT02637778.

**Supplementary Information:**

The online version contains supplementary material available at 10.1186/s12944-025-02500-1.

## Introduction

Cardiovascular diseases (CVD) are the leading cause of death globally. An estimated 17.9 million people died from CVDs in 2019, representing 32% of all global deaths [1, 2]. In 2021, the prevalence rate of CVD in Germany was 22.1% in people over 65 years of age. The highest rates of CVD were observed in the Federal States Saxony-Anhalt (27.6%), Saxony (26.8%) and Thuringia (27,0%) [3]. Because prevalence and mortality of CVD increase progressively with age, the demographic development of the European population may contribute to a further increase in the incidence of CVD and related costs for therapy [4]. Elevated total and LCL-C levels, hypertension and smoking are established CVD risk factors.

An increase of further specific markers such as fasting blood glucose, triglyceride levels, body mass index, and waist circumference further promotes the risk of cardiometabolic diseases, which represent one of the greatest global health challenges of the twenty-first century [5]. These diseases are responsible for disability-adjusted life years and mortality, thus there is a need for a coordinated global health initiative to fight against risk factors [6]. While sex, age, and genetic predisposition are beyond modification, most of the remaining cardiometabolic risk factors result from lifestyle with focus on diet [6]. Pörschmann et al. [7] analyzed the relationship between single dietary risk factors and cardiometabolic deaths in the World Health Organization European Region. The major drivers of estimated diet-related cardiometabolic deaths were a low intake of whole grains (326,755 deaths), followed by a diet low in legumes (232,918 deaths) and a diet high in sodium (193,713 deaths).

In accordance, in the *Nurses’ Health Study* and the *Health Professionals Follow-up Study*, higher intakes of polyunsaturated fatty acids (PUFA) and carbohydrates from whole grains were associated with a lower risk of coronary heart diseases (CHD; PUFA: hazard ratio (HR) 0.80, confidence interval (CI) [0.73 to 0.88], *p* < 0.001; carbohydrates from whole grains: HR 0.90, CI [0.83 to 0.98], *p* = 0.003) [8]. Replacing calories (5% of daily energy (En%)) from saturated fatty acids (SFA) with calories from monounsaturated fatty acids (MUFA), PUFA or dietary fibers were associated with a lower CHD risk (PUFA: HR 0.75, CI [0.67 to 0.84], *p* < 0.001; MUFA: HR 0.85, CI [0.74 to 0.97], *p* = 0.02; carbohydrates from whole grains (HR 0.91, CI [0.85 to 0.98]; *p* = 0.01).

Regular intake of 2–4 g long-chain n-3 PUFA such as eicosapentaenoic acid (EPA) and docosahexaenoic acid (DHA) per day resulted in a reduction of blood pressure and triglyceride (TG) concentrations [9–11]. Accordingly, highest concentrations of EPA, docosapentaenoic acid (DPA) and DHA in human tissues were associated with a 15–18% lower risk for death from all causes [12].

Higher intakes of soluble dietary fibers such as beta-glucan, found in oats, barley, edible mushrooms, and baker’s yeast, are associated with a reduction of total cholesterol (TC) and LDL-C [13–15]. Meta-analyses of prospective cohorts revealed significant associations between dietary fiber intake and a lower risk of all-cause mortality and mortality from CVD and CHD. A dose–response meta-analysis showed that an additional intake of 10 g/d dietary fiber was associated with a reduction in CVD mortality and CHD by 10–30% [16, 17].

To sum up, current data indicate the potential impact of dietary factors such as fat quality, carbohydrate quality and fiber intake on cardiometabolic health. Advice to encourage healthy or symptom-free individuals to consume a healthy diet to reduce their cardiometabolic risk at an advanced age is often ineffective because most efforts to change eating habits neglect the significance of motivation as well as the practicability in everyday life.

In sense with this, Fogacci et al. [18] formulated the need to translate the theoretical recommendations into practical concepts that consider everyday problems and are accessible to a wide range of people, regardless of socioeconomic status, including educational level. To address this gap, we developed a practice-oriented and science-based concept, named “**Mo**dulation **Ka**rdiovaskulärer **Ri**sikofaktoren” (MoKaRi). In particular, we integrated delicious recipes in personalized menu plans that helped the study participants to implement healthier eating in their daily life. To further increase compliance, various incentives and motivational strategies were included [19].

## Methods

### Study design

**Fig. 1:**
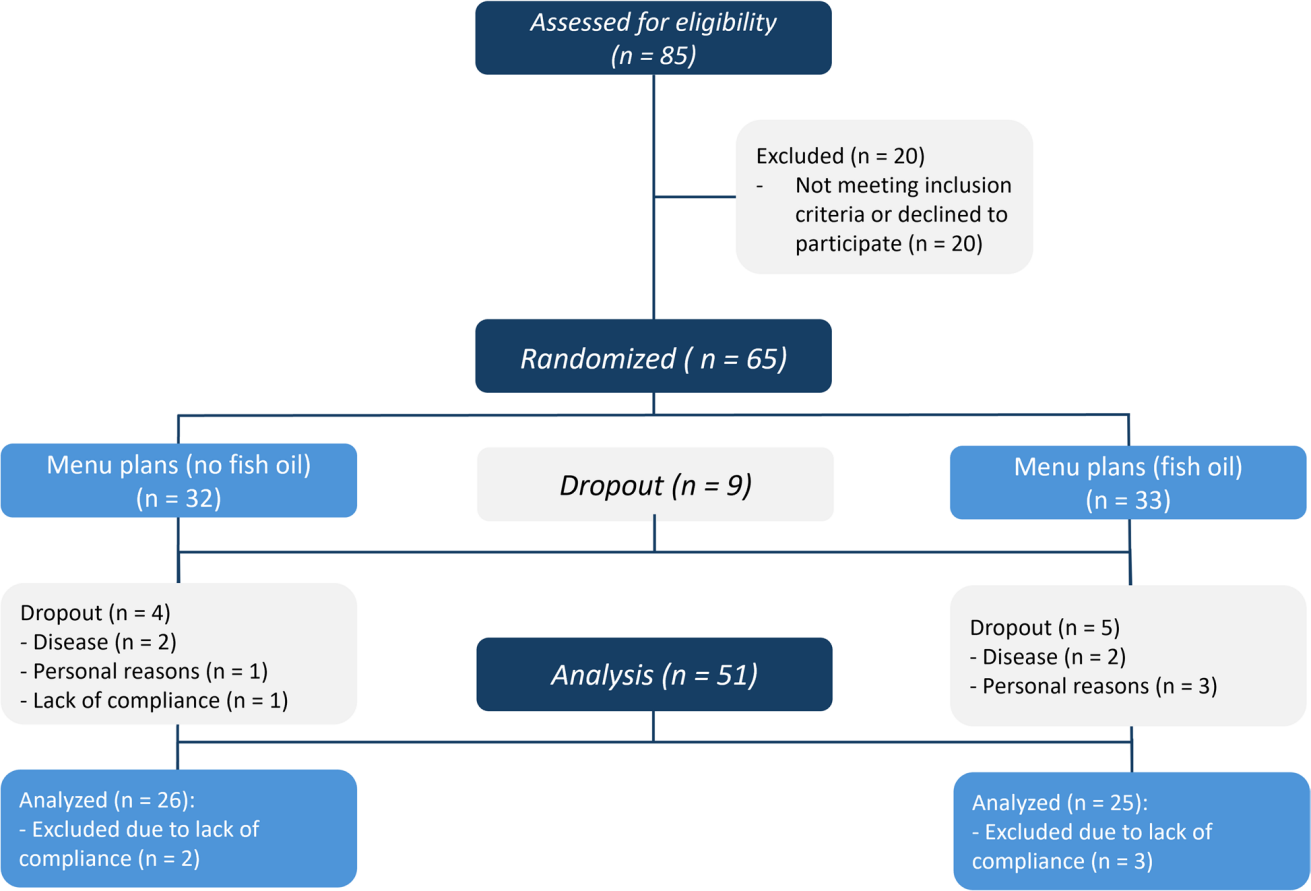
Flowchart diagram of the MoKaRi study. 85 subjects were screened for eligibility. 20 subjects had to be excluded, so that 65 subjects were randomized into the two intervention groups. After completion of the study, 26 and 25 participants were included in the statistical analyses

**Fig. 2:**
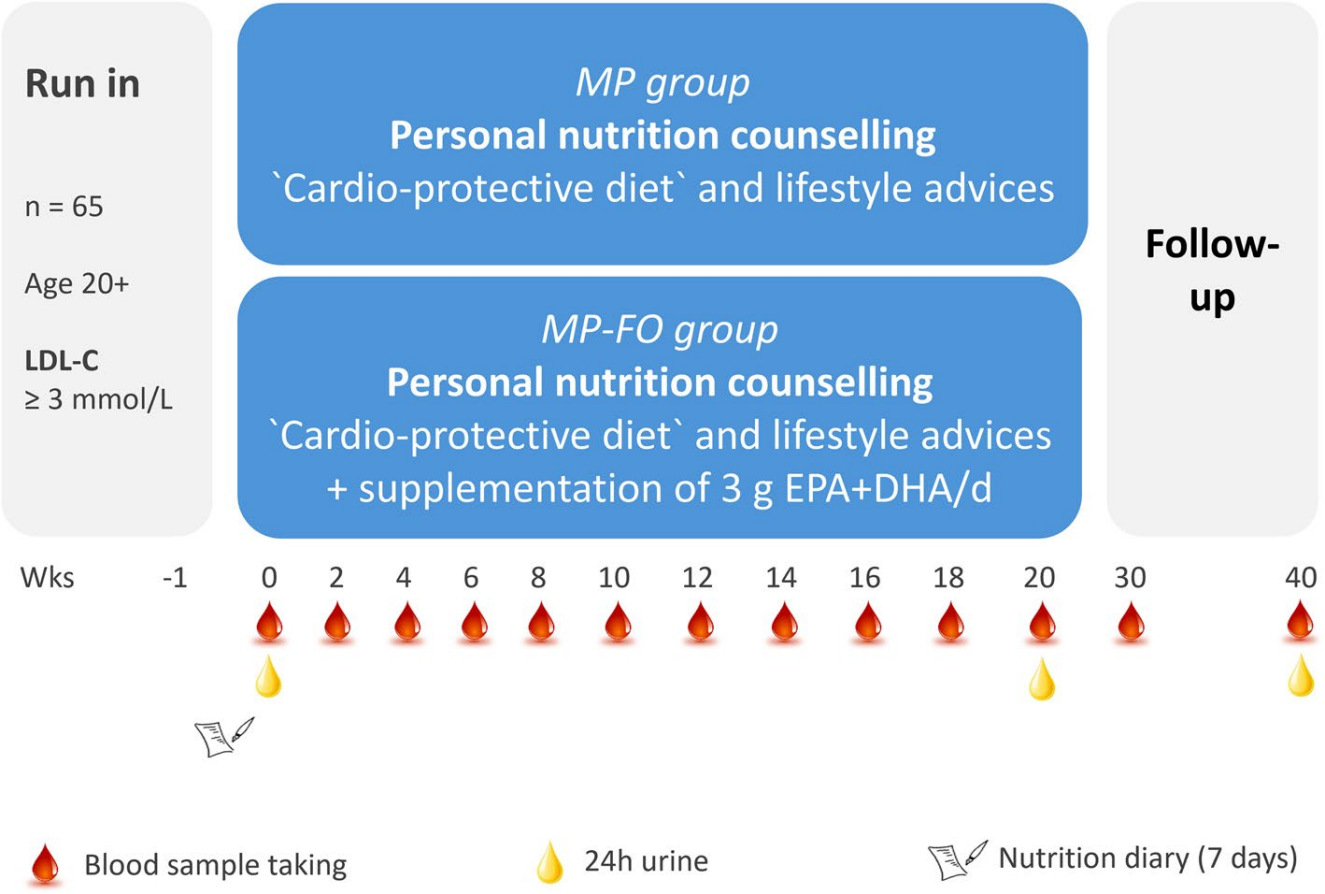
Design of the MoKaRi study. Abbreviations: DHA, docosahexaenoic acid; EPA, eicosapentaenoic acid; LDL-C, low-density lipoprotein cholesterol; MP, menu plan; MP-FO, menu plan plus fish oil

A total of 65 participants (LDL-C ≥ 3 mmol/l, without lipid-lowering therapy) from Thuringia, Germany were enrolled in the parallel-designed study (Fig. 1). The participants were randomly assigned to two groups. One group received the personalised daily menu plans, and the other group received the personalised daily menu plans plus 3 g EPA + DHA per day. Both interventions were isocaloric and based on personalized menu plans with optimized nutrient profiles for each day if the study (*n* = 140). In addition, personal nutritional counseling was provided every two weeks during the study period of 20 weeks (140 days) [19]. Incentives such as motivational talks, the provision of selected study foods, a sports programme (once a week), individual feedback on study parameters that reflect the state of health and group activities round off the MoKaRi concept. Blood samples were taken every two weeks during the 20-wk intervention and two times in the follow-up period (Fig. 2).

Participants of both sexes aged 20–80 years, and with plasma LDL cholesterol concentrations ≥ 3 mmol/L were eligible for inclusion of the MoKaRi study. The following exclusion criteria were determined: i) intake of lipid-lowering medications, glucocorticoids, drugs that interfere with glucose metabolism, ii) gastrointestinal diseases, known allergies or food intolerances, iii) known familial hypercholesterolemia, iv) intake of additional dietary supplements (e.g. fish oil capsules or vitamin E), v) pregnancy, lactation, and body mass index (BMI) ≥ 30 kg/m2 [19].

The primary outcome measure of the MoKaRi study was LDL-C [mmol/L] and the secondary outcome measures include further blood lipids, body weight, blood pressure, diabetes risk markers, vitamins, minerals and trace elements as published in [19].

Due to the study design, it was not possible to blind the participants to the intervention. The investigators, laboratory staff and statisticians were blinded until the conclusion of the data analysis.

The study protocol has been approved by the responsible ethical committee of the Friedrich Schiller University Jena, file number 4656–01/16). The study was registered before launch (ClinicalTrials.gov Identifier: NCT02637778). All inclusion and exclusion criteria and the complete study design has been published [19].

### Characteristics of the personalized menu plans (MoKaRi diet)

Our ‘cardioprotective diet’ (MoKaRi diet) based on menu plans which were prepared in dependence on individual needs. Each menu plan is characterized by (i) adequate intake of energy, carbohydrates, protein, and fat [20], (ii) appropriate intake of SFA (< 7 En%) [21], MUFA (> 10 En%) [20], PUFA (approx. 10 En%) [20] with a focus on EPA and DHA, (iii) encouraged consumption of vegetables and fruits, and (iv) intake of ≥ 40 g dietary fiber per day.

Menu plans for each day of the study were developed to reduce the intake of energy-dense food (rich in fat and SFA) and improve fat quality by replacing SFA with MUFA and n-3 PUFA. The intake of dietary fibers and further valuable nutrients, e.g. B vitamins, vitamin C, carotenoids, minerals, as well as EPA and DHA was optimized, and the intake of salt and simple sugars was reduced. The menu plans were adapted to individual energy requirements depending on age, sex, and physical activity. Half of the participants consumed an additional 3 g EPA + DHA per day. Further incentives, e.g. provision of selected study foods, regular feedback loops including discussions of parameters reflecting health status and cardiovascular risk, as well as activities encouraging group feeling (e.g. cooking courses), were implemented as part of the MoKaRi concept (for more details, see [19]).

### Anthropometric measurement, body composition, and blood pressure

Body weight, height, and waist circumference were measured with the fasting patient in light clothing. Body weight was measured using a calibrated scale with an integrated stadiometer (seca813; seca, Hamburg, Germany), and for waist circumferences, an ergonomic tape measure (seca212; seca, Hamburg, Germany) was used. Blood pressure was measured with a semiautomatic oscillometric device (boso-medicus uno; BOSCH + SOHN, Jungingen, Germany) with the fasting patient lying supine for at least 10 min in a temperature-controlled room (22–24 °C). Body composition was calculated by bioelectric impedance analysis (BIA 2000-S; Data Input, Pöcking, Germany).

### Sample collection and biochemical analyses

Between 7 and 10 a.m., fasting peripheral venous blood samples were collected and centrifuged (10 min, 2762 g, 4 °C) to separate plasma and serum. Urine was collected over 24 h prior to phlebotomy. Laboratory parameters such as blood lipids and blood count were analyzed immediately after blood sampling (Table S1). In addition, aliquots of serum, plasma, erythrocytes, and 24-h urine were obtained according to standard operation procedures and stored at −80 °C until analysis. Further secondary outcome measures such as biotin, vitamin B2, and vitamin C, blood lipids, lipoprotein(a) (Lp(a)), high-sensitive C-reactive protein (hs-CRP), homocysteine, clotting parameters, and minerals were measured as previously described (Table S1). Diabetes risk factors (fasting glucose and insulin) were analyzed and used to calculate the homeostatic model assessment for insulin resistance (HOMA-IR; Table S1). Blood collection, preparation of red blood cell fraction (RBC), lipid extraction and fatty acid (FA) analysis was carried out as described in Dittrich et al. [22].

### Food questionnaires

To record and document the subjects' eating behaviour and nutrient intake prior to the study, a complete self-report of individual food intake over seven days was compiled during the run-in phase of the MoKari study (Fig. 2). The dietary protocol used was based on the ‘Freiburg Dietary Protocol’ template, which was provided by PRODI® Version 6.4 (Nutri-Science, Stuttgart, Germany) and contains common foods and usual portion sizes. Foods that were not included in PRODI® were created by transferring the nutritional data from the packaging. The daily energy and nutrient intake was calculated using the PRODI® software package.

### Statistical analysis

This monocentric, randomized study examines the influence of personalized nutritional concepts on blood lipids over 20 weeks, with LDL-C as the primary outcome measure. The two-arm parallel design allows the comparison between the menu plan (MP) group (MoKaRi diet over 20 weeks) and the menu plan plus fish oil (MP-FO) group (MoKaRi diet plus 3 g EPA + DHA per day over 20 weeks; Fig. 2).

Our power calculation based on data by Jenkins et al. [23], who showed that a dietary intervention consisting of plant sterols, plant proteins, and fiber resulted in a reduction in LDL-C from 3.80 mmol/l (run-in) to 3.01 mmol/l [23]. Using nQuery version 7.0 (Statsols, Boston, United States), we estimated that 27 subjects are needed to provide an 80% power to a difference of 0.79 mmol/l (difference between μ1 = 3.80, μ2 = 3.01), assuming a standard deviation of 0.9 (using a 2-sided t-test with 0.05 as significance level).

Randomization into the two study groups was done using R version 4.0.1 (R Foundation for Statistical Computing, Vienna, Austria).

Data distributions were examined using the Shapiro–Wilk test. Differences between study groups were analyzed for normally distributed data using t-test for independent samples. Group comparisons were made using the Mann–Whitney U test for not-normally distributed data. The analyses of the changes over time within each study group were performed using ANOVA for repeated measurements (normally distributed data) or Friedman test (not-normally distributed data). For post-hoc testing, Fisher's Least Significant Difference test was performed, followed by a manual correction using the Benjamini–Hochberg procedure for multiple testing [24]. The presentation of the data (mean (± SD) and median (25th and 75th percentile)) is according to the statistical tests that were performed. Only data of subjects who attended every study appointment were included in all tests, except those where change from baseline calculations were performed. For the change from baseline tests, subjects only had to be present at the relevant times (baseline and week 20).

Correlation analyses were performed using Pearson correlation (normally distributed data) or Kendall’s rank correlation (not-normally distributed data).

## Results

### Baseline characteristics

Study participants were men (27%) and women (73%) aged 32–76 years at increased cardiovascular risk reflected by moderately elevated body mass index (BMI), TC, LDL-C, TG, fasting glucose, and blood pressure (Table 1). The random distribution between the two groups resulted in the following gender distribution: MP group: 9 men + 17 women / MP-FO: 6 men + 19 women.

**Table 1:** Baseline characteristics of the MoKaRi cohort (*n* = 56)

	**MP group**		**MP-FO group**		
**Parameter**	**Mean ± (SD)**	**Median** **(25th, 75th percentile)**	**Mean ± (SD)**	**Median** **(25th, 75th percentile)**	**p Value between groups**
Sex	9 m/17 w		6 m/19 w		
Age (years)	61 ± 11	64 (58, 69)	58 ± 12	62 (52, 68)	n.s
BMI (kg/m^2^)	28.9 ± 4.5	28.1 (25.8, 30.5)	28.5 ± 5.4	27.1 (25.0, 31.8)	n.s
TC (mmol/l)	7.1 ± 0.8	6.8 (6.6, 7.6)	6.6 ± 1.2	6.4 (5.7, 7.3)	n.s
HDL-C (mmol/l)	1.7 ± 0.4	1.6 (1.4, 1.9)	1.7 ± 0.4	1.6 (1.3, 2.0)	n.s
LDL-C (mmol/l)	4.8 ± 0.7	4.8 (4.3, 5.2)	4.3 ± 0.9	4.0 (3.6, 4.9)	0.015
Triglycerides (mmol/l)	1.7 ± 0.9	1.4 (1.2, 1.8)	1.6 ± 0.7	1.4 (1.2, 1.8)	n.s
Fasting glucose (mmol/l) (n^a^ = 51)	6.0 ± 1.0	5.7 (5.5, 6.2)	6.6 ± 3.9	6.0 (5.2, 6.4)	n.s
Insulin (mU/l)	10.7 ± 7.2	8.5 (7.0, 13.3)	8.2 ± 4.6	7.4 (5.5, 9.9)	n.s
HOMA-IR (n^a^ = 51)	2.8 ± 1.8	2.3 (1.6, 3.9)	2.4 ± 1.6	1.9 (1.4, 2.9)	n.s
HbA1c (%)	5.6 ± 0.5	5.6 (5.3, 6.0)	5.9 ± 1.5	5.6 (5.5, 5.8)	n.s
Systolic blood pressure (mm Hg)	143.3 ± 17.9	145.0 (132.3, 152.8)	140.7 ± 18.3	136.0 (128.0, 156.0)	n.s
Diastolic blood pressure (mm Hg)	82.0 ± 9.5	84.1 (78.9, 89.0)	84.3 ± 10.2	76.0 (73.0, 82.3)	n.s

*Abbreviations*: *BMI *body mass index, *HbA1c *glycated hemoglobin A_1c,_*HDL-C *high-density lipoprotein cholesterol, *HOMA-IR *homeostatic model assessment for insulin resistance, *LDL-C *low-density lipoprotein cholesterol, *TC *total cholesterol

^a^Number of data sets used to calculate shown data if differing from n.

The study participants recorded their intake of foods and drinks over a period of 7 days before the intervention. The calculated energy and nutrient intake did not differ between the two groups (Table 2). Compared with the recommendations for nutrient intake by the German Nutrition Society for adults (51–64 years), the average intakes of SFA, cholesterol, and sodium were higher, and the intakes of MUFA, PUFA, and potassium were lower than recommended (Table 2). In the run-in period, the intake of energy, carbohydrates, fibers, fat, in particular SFA and PUFA as well as long-chain n-3 PUFA, dietary cholesterol, sodium, and potassium differed markedly from the MoKaRi criteria and recommendations of the German Nutrition Society (Table 2).

**Table 2:** Energy and nutrient intakes by self-reports over 7 days before baseline in comparison with the recommendations of the German Nutrition Society and the MoKaRi criteria

**Energy and** **nutrients**	**DGE** **51–64 years**	**MoKaRi**	**MP group** **Characteristics**^a^	**MP-FO group** **Characteristics**^a^	p Value between groups
Energy (kcal/d)	W:1700 M: 2200	W:1700 M: 2200	2130 (1815, 2458)	2067 (1899, 2422)	n.s
Carbohydrate (g/d)	> 50 En%	> 50 En%	208 (162, 236) approx. 40 En%	235 (177, 261) approx. 46 En%	n.s
Fiber (g/d)	30	≥ 40	25.6 (22.2, 33.8)	30.6 (22.3, 34.8)	n.s
Total sugar (g/d)	n.a	/	101 (80, 133)	106 (88, 153)	n.s
Sucrose (g/d)	n.a	/	49.6 (31.6, 61.1)	46.5 (42.5, 75.9)	n.s
Glucose (g/d)	n.a	/	25.5 (17.5, 35.8)	26.3 (17.3, 38.5)	n.s
Fructose (g/d)	n.a	/	18.7 (15.4, 27.0)	21.3 (14.7, 25.9)	n.s
Protein (g/d)	0.8 g/kg body weight	> 15—20 En%	90 (73, 100) approx. 17 En%	84 (72, 94) approx. 16 En%	n.s
Fat (g/d)	30 En%	≤ 30 En%	85 (± 25) approx. 36 En%	89 (± 22) approx. 39 En%	n.s
SFA (g/d)	10 En%	≤ 7 En%	33.9 (± 11.6) approx. 14 En%	35.7 (± 9.3) approx. 16 En%	n.s
MUFA (g/d)	> 10 En%	≥ 10 En%	31.4 (± 10.8) approx. 14 En%	31.6 (± 8.2) approx. 14 En%	n.s
PUFA (g/d)	7–10 En%	≥ 10 En%	14.3 (9.8, 16.6) approx. 6 En%	13.6 (10.5, 17.9) approx. 6 En%	n.s
EPA (g/d)	0.25	EPA + DHA^∆^ 0.5 / 3.0	0.3 (0.1, 0.5)	0.2 (0.0, 0.4)	n.s
DHA (g/d)			0.3 (0.1, 0.6)	0.2 (0.1, 0.5)	n.s
Cholesterol (mg/d)	300	≤ 300	319 (± 126)	310 (± 116)	n.s
Potassium (mg/d)	4000	4000	3608 (3157, 4084)	3472 (2890, 4120)	n.s
Sodium (mg/d)	1500	1500	2411 (± 915)	2599 (± 884)	n.s

*Abbreviations*: *DGE *German Nutrition Society, *DHA *docosahexaenoic acid, *En% *% of daily energy, *EPA *eicosapentaenoic acid, *MP *menu plan, *MP-FO *menu plan plus fish oil, *MUFA *monounsaturated fatty acids, *n.a. *no data available, *n.s*. not significant, *PUFA *polyunsaturated fatty acids, *SFA *saturated fatty acids

^a^ Variables expressed as mean (± SD) or as median (25th, 75th percentile) depending on the statistical test that was performed; ∆ 0.5 g/d in the MP group, 3.0 g/d in the MP-FO group

### Effect of the MoKaRi diets on fatty acid distribution in erythrocyte lipids

Since the fatty acid distribution in the erythrocyte lipids reflects the dietary fat intake in the last two to three months, it can be used to assess the subjects' compliance with the dietary interventions.

In the MP group, there was an increase of oleic acid, linoleic acid (LA), alpha linolenic acid (ALA), DHA, total MUFA, and n-3 index after 20 weeks (*p* < 0.05; Table 3). This was also seen for EPA, DPA, DHA, and total n-3 PUFA and n-3 index in the MP-FO group (*p* < 0.05; Table 3). Simultaneously, we observed a decrease of arachidonic acid (AA), total SFA, and AA/DHA ratio in the MP group (*p* < 0.05; Table 3). In the MP-FO group oleic acid, AA, total MUFA, total n-6 PUFA, n-6/n-3 ratio, AA/EPA, and AA/DHA deceased significantly in the intervention period (Table 3). In the MP group, only AA/DHA ratio fell within the 20 weeks of the intervention period (*p* < 0.05; Table 3).

**Table 3:** Fatty acid distribution in erythrocyte lipids at baseline, after 10, 20, and 40 weeks of the MoKaRi study

**Parameter** **(% FAME)**	**wk**	**MP group** **(*n* = 26)** **Characteristics**^a^	p value within group	**MP-FO group** **(*n* = 25)** **Characteristics**^a^	p value within group	p value between groups
C-14:0	0	0.30 (0.27, 0.35)	a	0.28 (0.25, 0.32)	a,b	n.s
	10	0.19 (± 0.05) 0.18 (0.16, 0.22)	b	0.24 (± 0.07) 0.23 (0.20, 0.28)	c	0.006
	20	0.26 (0.24, 0.36)	a	0.26 (0.22, 0.33)	a,c	n.s
	40	0.19 (± 0.07) 0.18 (0.14, 0.20)	b	0.32 (± 0.10) 0.31 (0.24, 0.38)	b	< 0.001
	Cfb^∆^	−8.56 (± 30.04)		−4.10 (± 32.72)		n.s
C-15:0	0	0.16 (± 0.03) 0.16 (0.15, 0.18)	a	0.16 (± 0.04) 0.16 (0.13, 0.18)	a	n.s
	10	0.13 (± 0.02) 0.13 (0.11, 0.15)	b	0.15 (± 0.02) 0.16 (0.14, 0.16)	a	0.004
	20	0.17 (0.14, 0.17)	a	0.15 (0.13, 0.16)	a	n.s
	40	0.12 (0.10, 0.14)	b	0.15 (0.14, 0.16)	a	< 0.001
	Cfb^∆^	−0.96 (± 18.53)		−5.08 (± 17.39)		n.s
C-16:0	0	22.82 (± 1.61) 23.21 (21.87, 23.78)	a	24.25 (22.63, 25.20)	a	0.040
	10	20.98 (± 1.31) 21.09 (20.34, 21.55)	b	23.46 (22.77, 24.34)	a	< 0.001
	20	22.52 (± 1.26) 22.17 (21.55, 23.37)	a	23.63 (23.07, 24.41)	a	0.003
	40	19.12 (± 2.76) 18.59 (17.33, 20.96)	c	23.43 (23.03, 24.36)	a	< 0.001
	Cfb^∆^	−3.31 (−7.44, 1.05)		−1.93 (−7.04, 5.83)		n.s
C-17:0	0	0.26 (± 0.04) 0.25 (0.24, 0.28)	a,b	0.28 (± 0.05)	a,b	n.s
	10	0.26 (± 0.03) 0.25 (0.25, 0.28)	a	0.28 (± 0.03)	a	n.s
	20	0.27 (0.26, 0.30)	a	0.27 (± 0.03) 0.27 (0.26, 0.29)	a	n.s
	40	0.26 (± 0.04) 0.25 (0.24, 0.27)	b	0.26 (± 0.03)	b	n.s
	Cfb^∆^	6.71 (−2.85, 16.89)		4.88 (−5.63, 8.66)		n.s
C-18:0	0	11.22 (± 1.33) 10.92 (10.64, 12.16)	a,b	11.37 (10.64, 12.85)	a	n.s
	10	10.99 (± 0.89) 10.92 (10.33, 11.31)	a	10.52 (9.75, 11.20)	a	n.s
	20	11.65 (± 1.13)	b,c	10.65 (± 1.03) 10.61 (10.09, 11.14)	a	0.004
	40	12.22 (± 0.99) 12.14 (11.28, 12.79)	c	9.48 (8.91, 9.82)	b	< 0.001
	Cfb^∆^	5.52 (−9.87, 17.66)		−6.20 (−12.20, 0.89)		0.037
C-18:1 (n-9)	0	13.25 (± 1.96) 13.04 (12.47, 14.39)	a	16.16 (15.12, 16.67)	a	< 0.001
	10	15.59 (± 1.21) 15.45 (14.81, 16.01)	b	15.38 (14.52, 15.70)	b	n.s
	20	16.02 (± 1.16)	b,c	15.53 (± 0.95) 15.61 (14.90, 15.98)	b	n.s
	40	16.31 (± 1.62)	c	16.40 (± 1.11) 16.41 (15.63, 17.23)	a	n.s
	Cfb^∆^	19.14 (6.89, 34.57)		−3.54 (−7.40, −1.23)		< 0.001
C-18:2 (n-6, LA)	0	11.81 (± 1.50)	a	11.64 (± 2.03)	a	n.s
	10	11.90 (± 1.46)	a	9.60 (± 1.72)	b	< 0.001
	20	12.98 (± 1.73)	b	10.84 (± 1.58)	a	< 0.001
	40	12.77 (± 1.22)	b	13.32 (± 1.34)	c	n.s
	Cfb^∆^	9.92 (± 16.67)		−5.08 (± 18.08)		0.005
C-18:3 (n-3, ALA)	0	0.19 (± 0.04) 0.18 (0.16, 0.22)	a	0.18 (± 0.05)	a	n.s
	10	0.17 (± 0.04) 0.17 (0.14, 0.19)	a	0.16 (± 0.05)	a	n.s
	20	0.21 (± 0.04) 0.21 (0.19, 0.24)	b	0.19 (± 0.06)	a	n.s
	40	0.21 (0.19, 0.23)	b	0.23 (± 0.07) 0.21 (0.18, 0.27)	b	n.s
	Cfb^∆^	15.71 (1.07, 21.47)		−4.18 (−23.91, 38.09)		n.s
C-20:4 (n-6, AA)	0	13.14 (± 1.23) 13.25 (12.49, 13.90)	a	12.69 (11.71, 13.14)	a	n.s
	10	13.88 (± 1.70)	b	10.88 (± 1.57) 10.88 (9.87, 12.04)	a,c	< 0.001
	20	12.48 (± 1.46)	c	9.41 (± 1.49) 9.47 (8.10, 10.41)	b	< 0.001
	40	14.17 (± 1.97)	b	10.51 (± 1.49) 10.72 (10.21, 11.45)	c	< 0.001
	Cfb^∆^	−1.82 (−9.60, 2.49)		−26.03 (−33.38, −14.24)		< 0.001
C-20:5 (n-3, EPA)	0	1.03 (± 0.3) 0.97 (0.88, 1.14)	a	0.83 (± 0.33) 0.84 (0.56, 1.04)	a	n.s
	10	1.10 (0.96, 1.34)	a	4.09 (3.05, 4.82)	b	< 0.001
	20	1.06 (0.97, 1.36)	a	4.49 (3.20, 5.97)	b	< 0.001
	40	1.11 (0.90, 1.5)	a	1.36 (1.03, 2.26)	c	n.s
	Cfb^∆^	25.20 (± 44.55)		486.37 (± 311.87)		< 0.001
C-22:5 (n-3, DPA)	0	2.16 (± 0.27) 2.15 (1.95, 2.36)	a	1.88 (1.74, 2.02)	a	0.003
	10	2.39 (± 0.36)	b	2.89 (± 0.43) 2.95 (2.64, 3.10)	b	< 0.001
	20	2.08 (± 0.35)	a	3.10 (± 0.50) 3.14 (2.87, 3.44)	b	< 0.001
	40	2.58 (± 0.50)	b	2.45 (± 0.52) 2.35 (2.06, 2.67)	c	n.s
	Cfb^∆^	−1.14 (−9.54, 10.29)		65.70 (46.07, 80.56)		< 0.001
C-22:6 (n-3, DHA)	0	3.99 (± 0.91)	a	3.41 (± 0.98)	a	0.046
	10	5.25 (± 0.82)	b	5.58 (± 0.97)	b	n.s
	20	4.66 (± 0.76)	c	5.60 (± 0.83)	b	< 0.001
	40	5.34 (± 1.33)	b	4.47 (± 0.83)	c	0.012
	Cfb^∆^	21.85 (5.84, 43.48)		57.03 (42.85, 89.08)		< 0.001
Σ SFA	0	38.80 (± 1.48) 38.82 (38.21, 39.63)	a	38.78 (37.73, 40.58)	a,b	n.s
	10	33.92 (± 1.30) 33.78 (33.31, 34.36)	b	39.19 (38.30, 39.59)	a,b	< 0.001
	20	36.02 (± 1.57) 35.42 (35.11, 37.11)	c	39.05 (38.68, 40.06)	a	< 0.001
	40	32.65 (± 2.90)	b	38.58 (± 0.85) 38.51 (38.08, 39.19)	b	< 0.001
	Cfb^∆^	−8.52 (−11.21, −3.70)		1.65 (−3.04, 4.18)		0.002
Σ MUFA	0	15.73 (± 1.66)	a	18.19 (± 1.89) 18.62 (17.10, 19.29)	a	< 0.001
	10	17.59 (± 1.32) 17.49 (16.83, 18.07)	b	17.43 (16.73, 17.83)	b	n.s
	20	18.10 (± 1.24)	b,c	17.70 (± 0.95) 17.72 (17.14, 18.03)	b	n.s
	40	18.34 (± 1.75)	c	18.61 (± 1.20) 18.65 (17.93, 19.42)	a	n.s
	Cfb^∆^	15.13 (4.42, 26.83)		−4.43 (−6.51, −0.42)		< 0.001
Σ PUFA	0	36.94 (± 1.36) 37.11 (36.40, 37.55)	a	36.87 (34.62, 37.86)	a	n.s
	10	39.46 (± 1.92) 40.07 (38.68, 40.64)	b	36.79 (36.35, 37.54)	a	< 0.001
	20	37.66 (± 1.87)	a	36.42 (± 1.12) 36.20 (35.72, 37.44)	a	0.013
	40	41.36 (± 3.35)	b	36.67 (± 1.21) 36.58 (35.88, 37.37)	a	< 0.001
	Cfb^∆^	3.35 (0.42, 6.43)		−0.16 (−3.03, 3.20)		n.s
Σ n-6 PUFA	0	29.45 (28.41, 30.36)	a,b	29.48 (27.41, 30.64)	a	n.s
	10	30.62 (30.12, 31.45)	a,c	23.62 (21.97, 24.58)	b	< 0.001
	20	29.43 (± 1.70) 29.59 (28.85, 30.33)	b	22.65 (± 3.10) 22.52 (20.24, 23.97)	b	< 0.001
	40	31.79 (± 2.14) 32.35 (30.11, 33.87)	c	27.19 (± 1.91) 27.83 (25.77, 28.55)	a	< 0.001
	Cfb^∆^	−0.13 (−2.39, 5.15)		−23.56 (−31.05, −10.08)		< 0.001
Σ n-3 PUFA	0	7.56 (± 1.21)	a	6.41 (± 1.59) 6.65 (5.70, 7.30)	a	0.010
	10	9.04 (± 1.11)	b	12.57 (± 2.52) 13.34 (10.73, 14.41)	b	< 0.001
	20	8.15 (± 1.27)	a	13.54 (± 3.00) 13.97 (12.11, 16.00)	b	< 0.001
	40	9.48 (± 2.02) 9.50 (7.84, 10.81)	b	8.59 (7.48, 10.45)	c	n.s
	Cfb^∆^	14.66 (−1.19, 24.88)		112.28 (65.46, 141.18)		< 0.001
n-3 Index	0	5.02 (± 1.11) 5.04 (4.41, 5.77)	a	4.24 (± 1.25)	a	0.035
	10	6.40 (± 0.90) 6.44 (6.24, 6.84)	b	9.33 (± 2.15)	b	< 0.001
	20	5.85 (± 1.04) 5.89 (5.55, 6.28)	c	10.03 (± 2.56)	c	< 0.001
	40	6.26 (5.27, 7.50)	b,c	6.42 (± 1.96) 5.74 (5.04, 7.35)	d	n.s
	Cfb^∆^	24.32 (5.97, 35.75)		141.65 (81.44, 184.31)		< 0.001
n-6/n-3	0	3.97 (± 0.76) 3.95 (3.45, 4.38)	a	4.46 (± 0.75) 4.34 (4.18, 4.93)	a	0.038
	10	3.36 (3.08, 3.51)	b	1.75 (1.52, 2.39)	b	< 0.001
	20	3.77 (3.36, 3.94)	a,c	1.56 (1.29, 2.00)	c	< 0.001
	40	3.47 (± 0.62) 3.51 (3.14, 4.03)	b,c	3.11 (± 0.89) 3.24 (2.45, 3.82)	d	n.s
	Cfb^∆^	−12.22 (−19.17, 4.31)		−66.70 (−70.60, −50.58)		< 0.001
AA/EPA	0	13.87 (± 4.09) 14.04 (12.32, 15.22)	a	16.62 (± 6.47) 15.12 (12.47, 20.49)	a	n.s
	10	12.52 (10.53, 14.78)	a	2.59 (2.11, 3.92)	b	< 0.001
	20	12.15 (9.36, 13.29)	a	1.99 (1.40, 3.42)	b	< 0.001
	40	12.95 (± 4.69) 11.80 (9.89, 15.96)	a	7.80 (± 4.51) 8.11 (4.22, 10.71)	c	< 0.001
	Cfb^∆^	−17.18 (−35.03, −1.60)		−87.38 (−90.24, −76.38)		< 0.001
AA/DHA	0	3.19 (2.92, 3.98)	a	3.71 (3.20, 4.29)	a	n.s
	10	2.55 (2.35, 2.93)	b	1.97 (1.68, 2.30)	b	< 0.001
	20	2.64 (2.44, 2.83)	b	1.66 (1.28, 2.12)	c	< 0.001
	40	2.76 (± 0.56) 2.75 (2.51, 3.11)	b	2.46 (± 0.66) 2.56 (1.93, 2.97)	b	n.s
	Cfb^∆^	−24.15 (−30.70, −12.00)		−56.18 (−60.39, −43.35)		< 0.001

*Abbreviations*: *AA *arachidonic acid, *ALA *alpha linolenic acid, *Cfb *change from baseline, *CLA *conjugated linoleic acids, *DHA *docosahexaenoic acid, *DPA *docosapentaenoic acid, *EPA *eicosapentaenoic acid, *ETA *eicosatetraenoic acid, *FAME *fatty acid methyl ester, *LA *linoleic acid, *MUFA *monounsaturated fatty acids, *PUFA *polyunsaturated fatty acids, *SFA *saturated fatty acids, *TFA *trans fatty acids

^a^ Variables expressed as mean (± SD) and/or as median (25th, 75th percentile) depending on the statistical test that was performed; Cfb^∆^ Percentage change between baseline and the end of the intervention (week 20); Points in time without a common letter are significantly different, *p* < 0.05.

In the MP group, the amounts of C14:0, C15:0, C16:0, total SFA, n-6/n-3 ratio, and AA/DHA ratio decreased between baseline and follow up in week 40, whereas for C18:0, oleic acid LA, ALA, AA, DPA, DHA, total MUFA, total PUFA, total n-6 PUFA, total n-3 PUFA, n-3 index an increase was detected (p < 0.05; Table 3).

In the MP-FO group, we detected a reduction in C18:0, AA, n-6/n-3, AA/EPA, and AA/DHA over the 40 weeks (*p* < 0.05; Table 3). On the other site, we observed an increase in LA, ALA, EPA, DPA, DHA, total n-3 PUFA, and n-3 index in the MP-FO group (*p* < 0.05; Table 3).

**Fig. 3:**
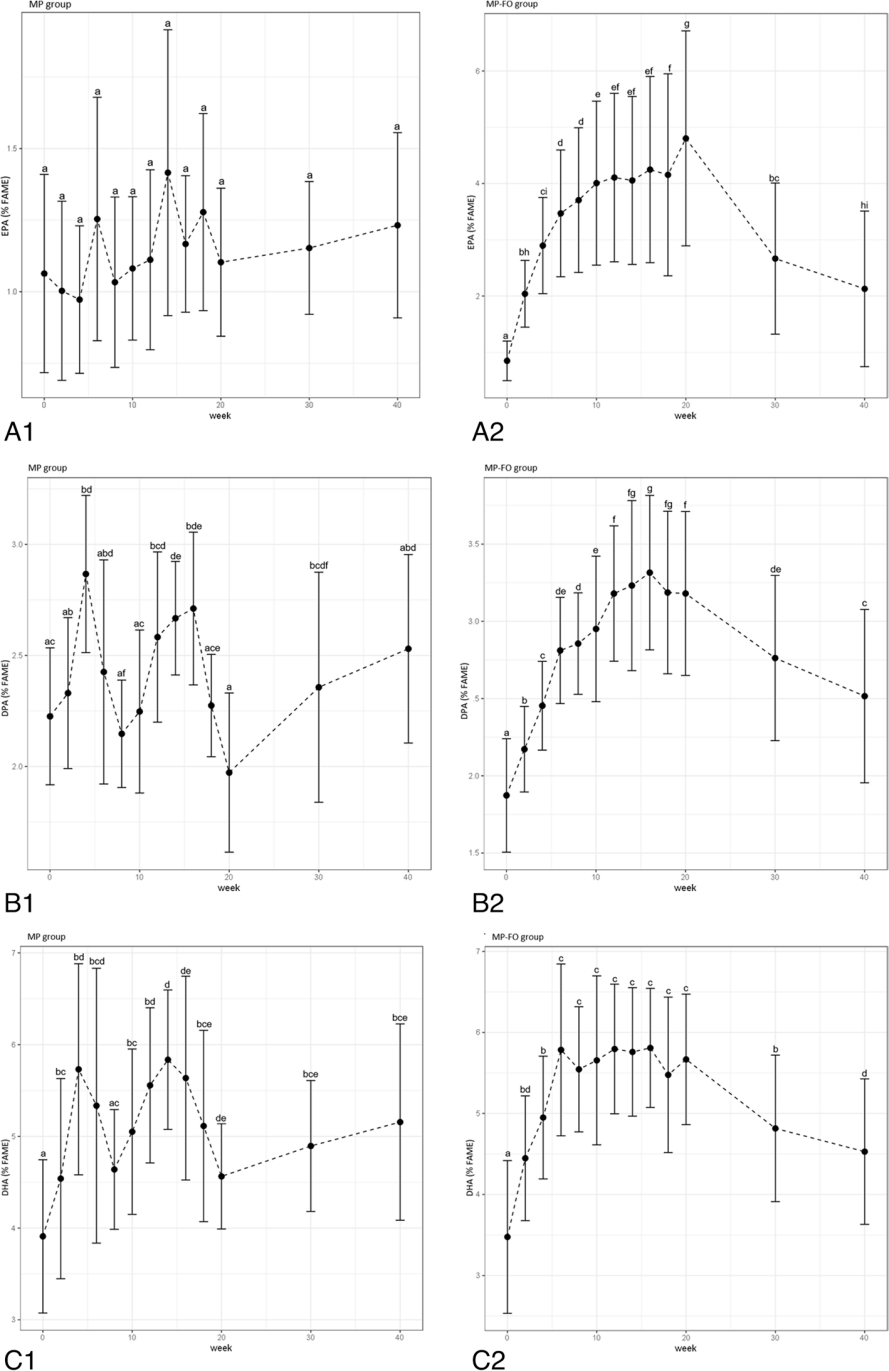
Change of (**A**) EPA, (**B**) DPA and (**C**) DHA in erythrocyte lipids (EL) over the study course in the MP group (Figure 3 A1-C1) and the MP-FO group (Figure 3 A2-C2). Abbreviations: DHA, docosahexaenoic acid; DPA, docosapentaenoic acid; EPA, eicosapentaenoic acid; EL, erythrocyte lipids; FAME, fatty acid methyl ester

The regular blood sampling every two weeks of the 20-week intervention period and twice in the follow-up period gives us a detailed insight into the extent of the incorporation of n-3 LC-PUFA into the EL. In the MP group, EPA and DPA varied during the intervention period, with no difference between baseline and values after 20 weeks (Fig. 3A1,B1). DHA also varied during the intervention period. An increase was observed after 20 weeks (Fig. 3C1).

In the MP-FO group, the fish oil supplementation was clearly reflected by changes in the fatty acids distribution of EL. For EPA, DPA, and DHA the higher concentrations measured after 2, 4, 6, 8, 1, 12, 14, 16, 18, and 20 weeks differed from baseline values (*p* < 0.05; Fig. 3A2-C2). In addition, the drop within the follow-up (weeks 30 and 40) was also evident for EPA, DPA, and DHA (*p* < 0.05; Fig. 3A2-C2). EPA increased gradually within the first 10 weeks and plateaued between week 10 and 16. A further increase was observed between weeks 18 and 20 (*p* < 0.05; Fig. 3A2). A steep increase in DPA was observed until week 16 (Fig. 3B2). The values fell marginally during the following 4 weeks. DHA increased until week 6 and then plateaued until week 20 (Fig. 3C2).

### Effect of the MoKaRi diets on the status of vitamins and minerals

For vitamins and minerals, the influence of the intervention with the MP was marginal (Table 4). In both groups, folic acid, vitamin B2, retinol, alpha-carotene, lutein + zeaxanthin, and lycopene remained constant over the study course (Table 4).

**Table 4:** Nutrient status – Vitamins, minerals, trace elements at baseline, after 10, 20, and 40 weeks of the MoKaRi study

**Parameter**	**wk**	**MP group (*n* = 26)** Characteristics*	*p* value within group	**MP-FO group (*n* = 25)** Characteristics*	*p* value within group		*p* value between groups
Biotin (ng/l)	0	377.4 (± 127.7) 338.0 (297.5, 445.5)	a,b	352.9 (± 135.0) 299.0 (258.5, 442.0)	a		n.s.
	10	405.0 (336.0, 532.5)	a	294.0 (248.8, 404.0)	a		n.s.
	20	331.0 (296.0, 505.5)	a,b	349.0 (234.5, 540.8)	a		n.s.
	40	318.0 (253.5, 486.0)	b	237.5 (172.0, 309.3)	b		0.039
	Cfb^∆^	−2.5 (−19.2, 31.0)		2.49 (−35.6, 35.7)			n.s.
Folic acid (ng/ml)	0	10.2 (± 4.3) 9.9 (6.9, 12.5)	a	9.7 (± 3.4) 8.7 (7.5, 12.7)	a		n.s.
	10	9.7 (7.6, 12.1)	a	8.7 (7.1, 11.3)	a		n.s.
	20	10.5 (7.7, 13.1)	a	8.4 (7.3, 12.1)	a		n.s.
	40	8.4 (6.9, 10.7)	a	9.5 (7.6, 11.6)	a		n.s.
	Cfb^∆^	4.5 (−6.9, 29.6)		−5.6 (−17.6, 21.7)			n.s.
Vitamin B_1_ (nmol/l)	0	134.5 (122.7, 145.1)	a,b	134.5 (115.6, 139.7)	a		n.s.
	10	132.1 (± 19.9) 129.2 (117.1, 144.1)	a	125.0 (± 17.1) 123.1 (114.9, 135.7)	a		n.s.
	20	128.4 (± 19.2) 126.3 (115.7, 138.4)	a	126.4 (± 19.7) 125.5 (112.7, 142.9)	a		n.s.
	40	154.5 (134.9, 163.9)	b	150.2 (142.9, 161.8)	b		n.s.
	Cfb^∆^	−3.6 (± 19.2)		−2.9 (± 15.2)			n.s.
Vitamin B_2_ (µg/l)	0	216.5 (± 39.1) 224.0 (199.0, 230.0)	a	237.9 (± 54.1)	a		n.s.
	10	238.0 (215.0, 271.5)	a	241.6 (± 37.5)	a		n.s.
	20	222.0 (209.0, 244.0)	a	235.3 (± 41.8)	a		n.s.
	40	255.0 (229.5, 270.5)	a	225.6 (± 66.1)	a		n.s.
	Cfb^∆^	7.6 (−1.5, 24.5)		2.0 (−10.7, 14.8)			n.s.
Vitamin B_6_ (nmol/l)	0	49.0 (37.6, 70.2)	a	53.2 (37.2, 72.5)	a		n.s.
	10	50.2 (35.3, 69.5)	a	66.2 (50.2, 84.9)	b		n.s.
	20	53.2 (38.0, 88.0)	a	65.3 (50.1, 83.1)	b		n.s.
	40	68.1 (48.8, 102.5)	a	53.6 (39.2, 70.4)	a		n.s.
	Cfb^∆^	16.6 (−7.8, 43.3)		34.7 (7.9, 70.7)			n.s.
Vitamin B_12_ (pmol/l)	0	271.0 (238.0, 312.0)	a	250.0 (221.0, 280.0)	a		n.s.
	10	248.0 (233.5, 303.5)	a	262.0 (225.5, 325.5)	a,b		n.s.
	20	282.3 (± 70.2) 279.0 (228.5, 334.0)	a	269.7 (± 91.8) 256.0 (207.5, 314.5)	a		n.s.
	40	331.2 (± 76.6) 317.0 (278.5, 392.5)	b	304.0 (± 86.9) 304.0 (237.5, 361.0)	b		n.s.
	Cfb^∆^	−1.9 (−17.0, 8.8)		2.8 (−10.7, 16.4)			n.s.
Holo-TC (pmol/l)	0	120.6 (101.2, 137.3)	a	110.2 (92.4, 146.4)	a		n.s.
	10	107.9 (88.3, 120.0)	a	111.9 (78.8, 125.6)	b		n.s.
	20	110.6 (96.8, 124.4)	a	101.20(79.1, 121.6)	b		n.s.
	40	121.6 (± 33.9) 118.8 (106.2, 142.1)	a	114.4 (± 29.2) 119.5 (98.3, 132.7)	a		n.s.
	Cfb^∆^	−8.3 (−21.1, 3.8)		−14.1 (−24.9, 1.4)			n.s.
Vitamin C (mg/l)	0	5.4 (3.7, 6.9)	a	4.4 (2.6, 5.2)	a	n.s.	
	10	5.2 (4.1, 6.9)	a	4.5 (3.3, 6.0)	a,b	n.s.	
	20	7.5 (± 3.5) 7.7 (4.4, 8.7)	a	8.3 (± 2.7) 8.8 (6.8, 9.6)	c	n.s.	
	40	7.1 (4.9, 10.1)	a	7.2 (4.3, 10.7)	b,c	n.s.	
	Cfb^∆^	39.1 (−30.8, 125.8)		66.7 (19.3, 183.9)		n.s.	
Retinol (µmol/L)	0	3.35 (± 0.89)	a	2.90 (± 0.49)	a	n.s.	
	10	3.24 (± 0.79)	a	2.67 (± 0.36)	a	0.018	
	20	3.06 (± 0.85)	a	2.80 (± 0.36)	a	n.s.	
	40	3.32 (± 0.71)	a	3.11 (± 0.68)	a	n.s.	
	Cfb^∆^	0.43 (−19.76, 10.28)		−3.69 (−11.77, 6.10)		n.s.	
alpha- carotene (µmol/L)	0	0.19 (0.14, 0.31)	a	0.27 (0.21, 0.44)	a	n.s.	
	10	0.22 (0.14, 0.35)	a	0.39 (0.20, 0.65)	a	n.s.	
	20	0.25 (0.15, 0.39)	a	0.49 (0.29, 0.67)	a	0.010	
	40	0.34 (0.21, 0.41)	a	0.50 (0.33, 0.61)	a	0.037	
	Cfb^∆^	8.56 (−17.68, 51.16)		41.61 (−4.26, 119.89)		n.s.	
beta-carotene (µmol/L)	0	0.41 (± 0.26) 0.33 (0.23, 0.59)	a	0.50 (0.31, 0.88)	a	n.s.	
	10	0.46 (± 0.21)	a	0.88 (± 0.59) 0.69 (0.50, 1.12)	a,b	0.021	
	20	0.48 (± 0.29) 0.36 (0.32, 0.62)	a	0.83 (0.62, 0.92)	b	0.004	
	40	0.50 (± 0.20) 0.46 (0.36, 0.61)	a	0.71 (0.57, 0.93)	b	0.008	
	Cfb^∆^	23.42 (−20.97, 64.79)		34.62 (11.28, 60.39)		n.s.	
Lutein plus zeaxanthin (µmol/L)	0	0.71 (0.54, 0.82)	a	0.81 (0.62, 1.18)	a	n.s.	
	10	0.77 (± 0.34) 0.71 (0.54, 0.88)	a	0.96 (± 0.35) 1.03 (0.76, 1.20)	a	n.s.	
	20	0.68 (± 0.26) 0.76 (0.48, 0.88)	a	0.94 (± 0.33) 0.90 (0.78, 1.15)	a	0.021	
	40	0.67 (0.58, 0.81)	a	1.02 (0.71, 1.25)	a	0.023	
	Cfb^∆^	−3.01 (−36.38, 22.94)		4.35 (−15.18, 34.04)		n.s.	
beta-crypto xanthin (µmol/L)	0	0.18 (± 0.12) 0.17 (0.07, 0.28)	a	0.39 (± 0.19) 0.37 (0.22, 0.56)	a	n.s.	
	10	0.14 (0.10, 0.19)	a	0.29 (0.18, 0.39)	a	0.001	
	20	0.12 (0.06, 0.18)	a	0.20 (0.16, 0.29)	b	0.005	
	40	0.13 (0.12, 0.18)	a	0.22 (0.18, 0.28)	b	0.014	
	Cfb^∆^	−24.79 (−55.26, 5.00)		−38.06 (−57.30, −17.13)		n.s.	
Lycopene (µmol/L)	0	0.13 (± 0.09) 0.11 (0.07, 0.18)	a	0.18 (± 0.11)	a	n.s.	
	10	0.11 (0.07, 0.24)	a	0.19 (± 0.11) 0.15 (0.12, 0.29)	a	n.s.	
	20	0.16 (± 0.09) 0.13 (0.09, 0.21)	a	0.25 (± 0.13)	a	0.020	
	40	0.11 (0.08, 0.22)	a	0.26 (± 0.14) 0.25 (0.13, 0.35)	a	0.015	
	Cfb^∆^	46.29 (−27.19, 134.28)		29.74 (11.57, 94.68)		n.s.	
Vitamin D (nmol/l)	0	49.0 (37.3, 65.3)	a	43.3 (28.1, 64.5)	a	n.s.	
	10	54.5 (40.1, 71.1)	a	42.0 (30.0, 52.0)	a	n.s.	
	20	63.5 (55.3, 78.4)	b	59.4 (45.1, 73.1)	b	n.s.	
	40	62.7 (54.7, 73.7)	b	60.5 (44.9, 72.6)	b	n.s.	
	Cfb^∆^	17.4 (7.9, 76.2)		34.6 (8.5, 55.7)		n.s.	
gamma- tocopherol (µmol/L)	0	3.1 (2.4, 3.3)	a,b	3.2 (± 0.6) 3.2 (3.0, 3.4)	a	n.s.	
	10	2.6 (2.2, 3.0)	a	2.6 (± 0.5) 2.5 (2.2, 3.0)	b	n.s.	
	20	2.8 (2.4, 3.1)	a,b	2.8 (± 0.7) 2.7 (2.3, 3.1)	b	n.s.	
	40	3.4 (± 1.1) 3.3 (2.5, 3.9)	b	3.2 (± 0.6)	a	n.s.	
	Cfb^∆^	−6.1 (± 18.7)		−10.9 (± 13.6)		n.s.	
alpha- tocopherol (µmol/L)	0	26.2 (± 11.9)	a	34.4 (± 8.5) 33.8 (28.5, 42.4)	a	0.036	
	10	25.1 (± 9.7) 23.2(18.8, 28.0)	a	22.9 (20.5, 30.6)	b,c	n.s.	
	20	23.8 (± 14.9) 22.0 (14.0, 30.9)	a	25.2 (22.1, 27.9)	c	n.s.	
	40	27.7 (± 12.8)	a	29.9 (± 9.0) 29.4 (24.5, 33.3)	a,b	n.s.	
	Cfb^∆^	−8.5 (−32.0, 13.2)		−27.5 (−40.6, −4.3)		n.s.	
Calcium (mmol/l)	0	2.5 (± 0.1)	a	2.4 (± 0.1)	a	n.s.	
	10	2.4 (± 0.1)	b	2.3 (± 0.1)	b	n.s.	
	20	2.4 (± 0.1)	b	2.3 (± 0.1)	b	n.s.	
	40	2.4 (± 0.1)	b	2.3 (± 0.1)	b	n.s.	
	Cfb^∆^	−3.5 (−6.0, −0.9)		−2.9 (−5.2, 0.0)		n.s.	
Potassium (mmol/l)	0	4.1 (3.9, 4.3)	a	4.1 (± 0.5) 4.2 (3.9, 4.2)	a	n.s.	
	10	3.9 (3.8, 4.2)	a	3.9 (± 0.3) 3.9 (3.7, 4.1)	b	n.s.	
	20	4.0 (± 0.3) 4.0 (3.8, 4.2)	a	4.0 (± 0.3)	a,b	n.s.	
	40	4.1 (± 0.4) 4.1 (3.8, 4.3)	a	4.0 (± 0.3)	a,b	n.s.	
	Cfb^∆^	−3.2 (−7.2, 0.2)		−3.1 (−6.4, 0.8)		n.s.	
Iron (µmol/l)	0	19.7 (15.9, 21.5)	a	18.7 (± 4.8) 18.5 (15.3, 20.9)	a	n.s.	
	10	15.3 (13.2, 18.5)	a	14.7 (± 4.8) 14.0 (10.8, 18.5)	b	n.s.	
	20	16.0 (14.6, 19.7)	a	15.1 (± 4.2) 14.7 (12.1, 16.4)	b	n.s.	
	40	17.8 (± 5.7) 17.9 (13.5, 20.2)	a	17.6 (± 4.5)	a	n.s.	
	Cfb^∆^	−12.8 (−34.3, 4.4)		−23.9 (−36.2, −1.1)		n.s.	
Ferritin (µg/l)	0	81.0 (52.5, 189.9)	a	87.5 (52.1, 125.3)	a	n.s.	
	10	64.3 (43.7, 111.8)	b	74.6 (35.5, 137.5)	a	n.s.	
	20	49.1 (33.8, 96.8)	c	64.4 (29.6, 105.0)	b	n.s.	
	40	66.0 (42.9, 114.9)	b,c	72.9 (39.0, 114.5)	b	n.s.	
	Cfb^∆^	−37.2 (−52.3, −27.2)		−36.8 (−48.5, −17.3)		n.s.	
Transferrin (g/l)	0	2.8 (± 0.5)	a	2.8 (± 0.4)	a	n.s.	
	10	2.6 (± 0.4)	a	2.7 (± 0.4)	a	n.s.	
	20	2.7 (± 0.5)	a	2.7 (± 0.3)	a	n.s.	
	40	2.7 (± 0.4)	a	2.7 (± 0.3)	a	n.s.	
	Cfb^∆^	0.3 (± 7.0)		0.1 (± 7.6)		n.s.	
Iodine (µg/l)	0	52.4 (48.5, 57.7)	a,b	58.1 (± 11.6) 56.4 (48.6, 67.5)	a,b	n.s.	
	10	59.7 (49.0, 72.4)	a	64.2 (± 13.5) 61.5 (54.2, 73.1)	a	n.s.	
	20	49.3 (± 10.7) 49.2 (44.9, 54.7)	b	54.9 (± 9.6)	b	n.s.	
	40	57.1 (± 9.2) 55.6 (51.5, 62.2)	a	65.6 (± 15.6)	a	0.048	
	Cfb^∆^	−4.6 (± 19.4)		−4.0 (± 17.5)		n.s.	
Creatinine 24h urine (mmol/24h)	0	10.0 (± 5.3) 8.8 (6.0, 11.8)	a	10.3 (± 3.1) 9.8 (7.9, 11.9)	a	n.s.	
	20	10.6 (± 4.1) 10.1 (8.3, 13.5)	a	9.5 (± 2.6) 8.8 (7.9, 11.1)	a	n.s.	
	40	4.6 (3.6, 6.1)	b	5.2 (3.7, 6.9)	b	n.s.	
	Cfb^∆^	−1.1 (−18.0, 18.3)		−4.7 (−20.5, 8.6)		n.s.	
Methylmalonic acid 24 h urine (mmol/24h)	0	1.3 (± 0.6) 1.3 (1.0, 1.7)	a	1.6 (± 0.6) 1.5 (1.3, 2.0)	a	n.s.	
	20	1.6 (± 0.6) 1.7 (1.1, 2.1)	a	1.8 (± 0.7) 1.7 (1.3, 2.4)	a	n.s.	
	40	1.6 (1.1, 2.1)	a	1.8 (1.1, 2.2)	a	n.s.	
	Cfb^∆^	17.8 (−25.2, 60.2)		13.4 (−8.4, 40.0)		n.s.	
Magnesium 24h urine (mmol/24h)	0	4.1 (± 2.0 4.1 (2.3, 5.7)	a	4.2 (± 1.5) 4.2 (3.3, 5.3)	a	n.s.	
	20	4.2 (3.3, 5.4)	a	4.0 (3.2, 4.7)	a	n.s.	
	40	4.7 (± 2.3) 4.4 (3.2, 6.4)	a	4.4 (± 1.4) 4.6 (3.5, 5.4)	a	n.s.	
	Cfb^∆^	7.7 (−12.2, 38.6)		−0.7 (−21.0, 16.0)		n.s.	
Sodium 24h urine (mmol/24h)	0	116.2 (± 50.0) 114.5 (79.0, 149.0)	a	137.6 (± 47.1)	a	n.s.	
	20	142.5 (88.3, 186.8)	a	121.9 (± 48.8) 121.0 (85.0, 148.0)	a,b	n.s.	
	40	80.7 (± 31.1) 80.0 (60.5, 102.8)	b	90.4 (± 50.7)	b	n.s.	
	Cfb^∆^	6.5 (−21.4, 47.6)		0.33 (−44.7, 17.2)		n.s.	
Selenium 24h urine (mmol/24h)	0	0.16 (0.09, 0.19)	a	0.19 (0.14, 0.23)	a	n.s.	
	20	0.21 (0.16, 0.26)	a,b	0.23 (0.20, 0.31)	a	n.s.	
	40	0.27 (± 0.11) 0.24 (0.19, 0.35)	b	0.30 (± 0.15) 0.29 (0.18, 0.36)	a	n.s.	
	Cfb^∆^	72.37 (± 110.47)		73.12 (± 106.58)		n.s.	
Zinc 24h urine (mmol/24h)	0	6.8 (2.8, 7.6)	a	6.8 (4.1, 10.6)	a	n.s.	
	20	6.3 (3.5, 9.3)	a	5.5 (4.2, 9.3)	a	n.s.	
	40	7.2 (3.7, 12.2)	a	6.6 (4.5, 8.2)	a	n.s.	
	Cfb^∆^	14.9 (−16.5, 31.3)		−7.6 (−36.0, 28.0)		n.s.	

*Abbreviations*: *Cfb *change from baseline, *Holo-TC *holo-transcobalamin

^*^ Variables expressed as mean (± SD) and/or as median (25th, 75th percentile) depending on the statistical test that was performed; Cfb^∆^ Percentage change between baseline and the end of the intervention (week 20); Points in time without a common letter are significantly different, *p* < 0.05.

Within the intervention period, an increase in vitamin B_6_, vitamin C, beta-carotene, vitamin D was observed in the MP-FO group whereas in the MP group only vitamin D increased (*p* < 0.05; Table 4). Concomitant we found a decrease in holo-transcobalamin (holo-TC), beta-cryptoxanthin, alpha- and gamma-tocopherol, calcium, iron, and ferritin in the MP-FO group (*p* < 0.05; Table 4). This was also seen for calcium, and ferritin in the MP group (*p* < 0.05; Table 4).

After the follow-up period, biotin, beta-cryptoxanthin, calcium, ferritin, creatinine, and sodium were below the baseline values in the MP-FO group (*p* < 0.05; Table 4). This was also evident for calcium, ferritin, creatinine, and sodium in the MP group (*p* < 0.05; Table 4). Contrary, vitamin B_1_, vitamin B_12_, beta-carotene, and vitamin D increased from baseline to follow-up in the MP-FO group (*p* < 0.05; Table 4). In the MP group, this increase was also observed for vitamin B_12_, vitamin D, and selenium (*p* < 0.05; Table 4).

### Effect of the MoKaRi diets on body weight and body composition

Although the MoKaRi diets were not hypocaloric, body weight, BMI, and waist circumference decreased significantly in both groups, whereby the more pronounced reduction in body weight and BMI in the MP-FO group was significantly larger than the decrease in the MP group (*p* < 0.05). The changes achieved were maintained over the follow-up. Body fat dropped after 10 weeks of intervention in both groups (*p* < 0.05). While body fat levels in the MP-OF group continued to decrease to the end of the intervention (*p* < 0.05), the reduction in the MP group failed significance at week 20 (Table 5).

**Table 5:** Anthropometric measurements and body composition after 10, 20, and 40 weeks of the MoKaRi study

**Parameter**	**wk**	**MP group (*n* = 26)**	*p* Value within group	**MP-FO group (*n* = 25)**	*p* Value within group	*p* Value MP vs. MP-FO
		Characteristics*		Characteristics*		
Body weight (kg)	0	81.0 (± 13.1)	a	80.1 (± 17.0) 78.5 (67.3, 89.4)	a	0.837
	10	78.1 (± 12.5)	b	75.8 (± 16.4) 73.3 (63.9, 83.9)	b	0.590
	20	76.5 (± 11.9)	c	73.8 (± 17.5) 70.4 (62.1, 83.7)	c	0.529
	40	77.1 (± 12.3) 74.5 (70.6, 82.4)	b,c	68.7 (61.9, 84.4)	b,c	0.293
	Cfb^∆^	−5.1 (± 4.1)		−8.3 (± 6.0)		0.034*
BMI (kg/m^2^)	0	28.9 (± 4.5) 28.8 (25.8, 30.5)	a	28.5 (± 5.4) 27.1 (25.0, 31.8)	a	0.758
	10	27.9 (± 4.4) 27.2 (24.8, 29.4)	b	27.0 (± 5.0) 25.1 (23.2, 29.2)	b	0.479
	20	26.2 (24.5, 29.5)	b	25.3 (23.0, 28.6)	c	0.266
	40	26.9 (25.1, 28.7)	b	25.1 (22.8, 28.7)	b,c	0.208
	Cfb^∆^	−5.1 (± 4.1)		−8.3 (± 6.0)		0.034*
Waist circumferences (cm)	0	99.1 (± 11.3)	a	99.2 (± 14.7) 98.0 (89.3, 106.8)	a	0.992
	10	94.5 (± 7.0) 91.0 (87.0, 100.0)	b	91.0 (82.3, 96.8)	b	0.319
	20	93.0 (± 9.6) 91.0 (86.0, 97.0)	b,c	89.5 (80.0, 96.5)	b	0.301
	40	92.4 (± 10.1)	c	90.0 (± 12.1) 87.5 (80.8, 94.8)	b	0.472
	Cfb^∆^	−5.8 (± 3.8)		−8.5 (± 6.7)		0.096
Body fat (kg)	0	25.0 (± 8.1)	a	24.2 (± 10.8) 21.6 (17.0, 27.7)	a	0.799
	10	22.6 (± 8.2)	b	21.2 (± 10.0) 17.9 (15.3, 25.1)	b	0.644
	20	22.5 (± 8.1) 22.6 (17.5, 26.8)	a,b	16.4 (11.5, 25.1)	c	0.156
	40	23.6 (± 7.1) 22.9 (20.0, 26.4)	a,b	18.6 (15.3, 25.0)	a,b	0.257
	Cfb^∆^	−11.2 (−27.1, −4.0)		−20.0 (−26.9, −9.9)		0.123
Body fat (%)	0	30.7 (± 7.9)	a	29.8 (± 8.4)	a	0.733
	10	28.8 (± 8.6)	b	27.3 (± 8.5)	b	0.607
	20	28.5 (± 8.2)	a,b	24.9 (± 9.1)	c	0.225
	40	30.0 (± 7.4)	a,b	28.5 (± 8.4)	a	0.568
	Cfb^∆^	−6.6 (−19.1, −3.3)		−13.6 (−21.4, −5.3)		0.207
Body water (l)	0	38.6 (34.5, 47.9)	a	39.1 (34.2, 44.3)	a	0.802
	10	39.9 (± 6.7) 37.7 (35.4, 46.4)	a	39.5 (± 7.9) 38.0 (33.9, 44.3)	a	0.872
	20	39.3 (36.2, 46.5)	a	36.9 (33.1, 43.4)	a	0.452
	40	37.9 (33.8, 46.3)	a	35.4 (32.7, 42.4)	b	0.315
	Cfb^∆^	−0.7 (−1.6, 1.6)		−1.9 (−4.1, 1.1)		0.085
Lean body mass (kg)	0	55.5 (47.1, 65.6)	a	53.4 (46.7, 60.5)	a	0.639
	10	55.3 (± 9.6) 53.6 (48.4, 64.3)	a	53.9 (± 10.8) 51.9 (46.2, 60.5)	a	0.680
	20	53.7 (49.5, 63.5)	a	50.3 (45.2, 59.3)	a	0.433
	40	51.8 (46.1, 63.3)	a	48.4 (44.7, 57.9)	b	0.315
	Cfb^∆^	−1.0 (−2.1, 1.6)		−1.9 (−4.1, 1.1)		0.149

*Abbreviations*: *BMI *body mass index, *MP *menu plan, *MP-FO *menu plan plus fish oil; * p ≤ 0.05

^*^ Variables expressed as mean (± SD) and/or as median (25th, 75th percentile) depending on the statistical test that was performed; Cfb^∆^ Percentage change between baseline and end of intervention (week 20); times without a common letter are significantly different, *p* < 0.05.

### Effect of the MoKaRi diets on blood lipids

**Fig. 4:**
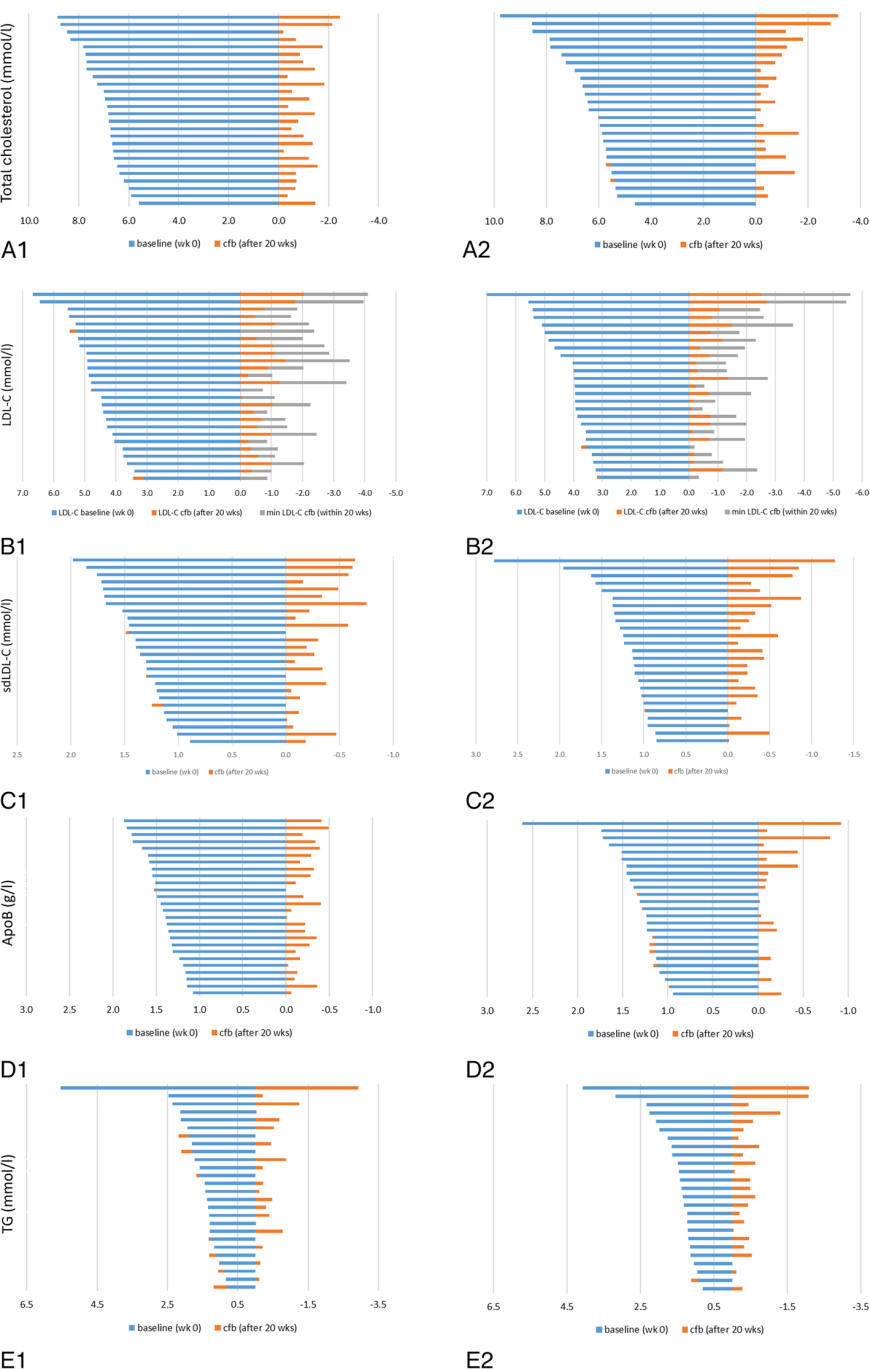
Concentrations at baseline (week 0) and after 20 weeks (change from baseline cfb) for each individual participant in the MP group (left) and the MP-FO group (right)—**A1-2:** Total cholesterol (TC, mmol/l), **B1-2**: LDL-C and min LDL-C (mmol/l), **C1-2**: sdLDL-C (mmol/l), **D1-2**: apolipoprotein B (mg/l), **E1-2**: TG (mmol/l)

Our primary outcome measure LDL-C and further associated parameters were significantly reduced in both groups (*p* < 0.05; Table 6). The significant reduction on TC, LDL-C, sdLDL-C, apolipoproteins B, and TG (*p* < 0.05 only in the MP-FO group) after the 20-week intervention were observed in almost all test subjects and the reduction seems to increase with higher initial values (Table 6; Fig. 4A-E).

**Table 6:** Blood lipids at baseline after 10, 20, and 40 weeks of the MoKaRi study

**Parameter**	**wk**	**MP group** **(*n* = 26)** Characteristics*	p Value within group			**MP-FO group** **(*n* = 25)** Characteristics*	p Value within group	p Value MP vs. MP-FO
TC (mmol/l)	0	7.1 (± 0.8)	a			6.6 (± 1.2)	a	0.092
	10	6.0 (± 0.8)	b			5.8 (± 0.8)	b,c	0.590
	20	6.2 (± 0.8)	b			5.8 (± 0.8)	b	0.126
	40	6.5 (± 1.0)	c			6.2 (± 1.0)	a,c	0.245
	Cfb^∆^	−14.5 (± 7.9)				−11.3 (± 10.7)		0.228
Non-HDL-C (mmol/l)	0	5.4 (± 0.7) 5.4 (5.0, 5.7)	a			4.6 (4.1, 5.7)	a	0.026*
	10	4.4 (± 0.7)	b			4.3 (± 0.7) 4.3 (3.9, 4.8)	b	0.845
	20	4.6 (± 0.7)	b			4.3 (± 0.8) 4.3 (3.7, 4.7)	b	0.124
	40	5.0 (± 0.9)	c			4.6 (± 1.0) 4.5 (4.0, 5.0)	a,b	0.191
	Cfb^∆^	−16.4 (± 9.9)				−12.5 (± 13.1)		0.229
HDL-C (mmol/l)	0	1.7 (± 0.4)	a			1.7 (± 0.4)	a	0.769
	10	1.6 (± 0.4)	b			1.5 (± 0.4)	b	0.424
	20	1.6 (± 0.3)	b			1.5 (± 0.3)	b,c	0.639
	40	1.6 (± 0.3)	b			1.6 (± 0.4)	c	0.906
	Cfb^∆^	−6.2 (± 7.8)				−6.2 (± 11.2)		0.990
Apolipoprotein A1 (g/l)	0	1.9 (± 0.3)	a			1.9 (± 0.3) 1.9 (1.8, 2.0)	a	0.949
	10	1.6 (± 0.4)	b			1.5 (± 0.2) 1.5 (1.4, 1.6)	b	0.288
	20	1.6 (± 0.3) 1.6 (1.4, 1.8)	b			1.6 (1.5, 1.7)	b	0.194
	40	1.6 (± 0.3)	b			1.7 (± 0.4) 1.7 (1.4, 1.9)	c	0.479
	Cfb^∆^	−14.9 (± 5.7)				−19.6 (± 6.5)		0.009**
LDL-C (mmol/l)	0	4.8 (± 0.7) 4.8 (4.3, 5.2)	a			4.0 (3.6, 4.9)	a	0.015*
	10	3.8 (± 0.6)	b			3.5 (± 0.6) 3.5 (3.3, 3.8)	b	0.095
	20	4.1 (± 0.6)	c			3.6 (± 0.6) 3.7 (3.2, 3.8)	b,c	0.007**
	40	4.4 (± 0.8)	d			3.9 (± 0.8) 3.9 (3.3, 4.3)	c	0.029*
	Cfb^∆^	−14.3 (± 10.9)				−15.8 (± 13.3)		0.676
	0	1.4 ± 0.3 1.4 (1.2, 1.6)	a			1.1 (1.0, 1.4)	a	0.033*
	10	± 0.3 1.1 (0.9,1.2)	b			0.9 (0.8, 1.0)	b	0.005**
sdLDL-C (mmol/l)	20	1.1 ± 0.2 1.2 (1.0, 1.3)	b,c			0.9 (0.7, 1.1)	b,c	0.001**
	40	1.2 ± 0.2 (1.1, 1.4)	c,d			1.1 (0.9, 1.5)	a,d	0.391
	Cfb^∆^	−18.1 (± 15.1) −17.0 (−30.4, −6.5)				−27.0 (± 17.8) −24.1 (−38.1, −11.9)		0.077
Apolipoprotein B (g/l)	0	1.5 (± 0.2) 1.4 (1.3, 1.6)	a			1.3 (1.1, 1.4)	a	0.030*
	10	1.2 (± 0.2)	b			1.2 (± 0.2) 1.3 (1.2, 1.4)	a,b	0.644
	20	1.2 (± 0.1)	b			1.2 (± 0.2) 1.2 (1.1, 1.3)	b	0.694
	40	1.3 (± 0.2)	c			1.3 (± 0.3) 1.3 (1.1, 1.5)	a,b	0.667
	Cfb^∆^	−14.7 (−20.6, −8.5)				−5.80 (−14.56, 1.02)		0.040*
Triglycerides (mmol/l)	0	1.4 (1.2, 1.8)		a	1.4 (1.2, 1.8)		a	0.900
	10	1.3 (0.9, 1.6)		a	1.2 (0.9, 1.3)		b,c	0.285
	20	1.3 (1.0, 1.4)		a	1.0 (0.9, 1.4)		b	0.114
	40	1.2 (1.1, 1.4)		a	1.2 (1.0, 2.2)		a,c	0.847
	Cfb^∆^	−14.0 (−29.6, 2.1)			−27.8 (−42.3, −15.1)			0.040*
Lipoprotein(a) (mg/l)	0	171.0 (52.0, 311.0)		a	97.0 (46.0, 227.5)		a	0.481
	10	156.0 (54.0, 251.0)		a	99.0 (48.0, 232.5)		a	0.741
	20	155.0 (55.0, 310.0)		a	101.0 (47.8, 245.8)		a	0.641
	40	173.0 (54.0, 338.0)		a	105.0 (48.3, 222.5)		a	0.633
	Cfb^∆^	−0.6 (−13.2, 6.0)			7.4 (−3.4, 20.7)			0.068

*Abbreviations*: *HDL-C *high-density lipoprotein cholesterol, *LDL-C *low-density lipoprotein cholesterol, *MP *menu plan, *MP-FO *menu plan plus fish oil, *TC *total cholesterol; * *p* ≤ 0.05; ** *p* ≤ 0.01

* Variables expressed as mean (± SD) and/or as median (25th, 75th percentile) depending on the statistical test that was performed; Cfb∆ Percentage change between baseline and end of intervention (week 20); times without a common letter are significantly different, *p* < 0.05.

In detail, a reduction of baseline TC, high-density lipoprotein cholesterol (HDL-C), LDL-C and non-HDL-C concentrations was observed after 10 and 20 weeks of intervention in both groups (*p* < 0.05). Except for TC and non-HDL-C in the MP-FO group, the decrease of baseline values remained significant after the follow-up (*p* < 0.05; Table 6).

After 20 weeks of intervention, baseline concentrations of apolipoprotein A1 and HDL-C decreased in both groups (*p* < 0.05). The more substantial reduction of apolipoprotein A1 in the MP-FO group differed from the smaller decrease in the MP group (−20% vs. −15%; *p* < 0.05).

LDL-C concentrations decreased in both groups after 10 weeks and a further reduction was observed after 20 weeks of the intervention (*p* < 0.05; Table 6). In the MP-FO group, LDL-C was lower at baseline, after the 20-week intervention and after the follow-up compared to the MP group (*p* < 0.05). The small dense LDL-C (sdLDL-C) concentrations were calculated as described by Srisawasdi et al. [25], and a notable decline was observed in both groups after 10 and 20 weeks, with a more pronounced reduction evident in the MP-FO group (−27% vs. −17%; Table 6).

To rule out the possibility that the decrease in TC and LDL-C was due to weight loss, we performed a correlation analysis, which showed no association between absolute changes in body weight and changes in TC (r −0.011; *p* = 0.907) or LDL-C (r 0.045, *p* = 0.645).

After 20 weeks, apolipoprotein B decreased also in both groups, whereas the extend was higher in the MP group (−15% vs −6%; *p* < 0.05; Table 6). In our data, we found a strong correlation between apolipoprotein B and LDL-C (r 0.859, *p* < 0.001).

The decrease of TG in the MP group was not significant. In the MP-FO group, TG dropped after 10 and 20 weeks of intervention (*p* < 0.05). The reduction in TG was twice as high in the MP-FO as in the MP group after 20 weeks of intervention (−28% vs. −14%; *p* < 0.05; Table 6).

Lipoprotein(a) always remained unchanged (Table 6).

### Changes in body weight and LDL-C at two-week intervals during the 20-week intervention

**Fig. 5:**
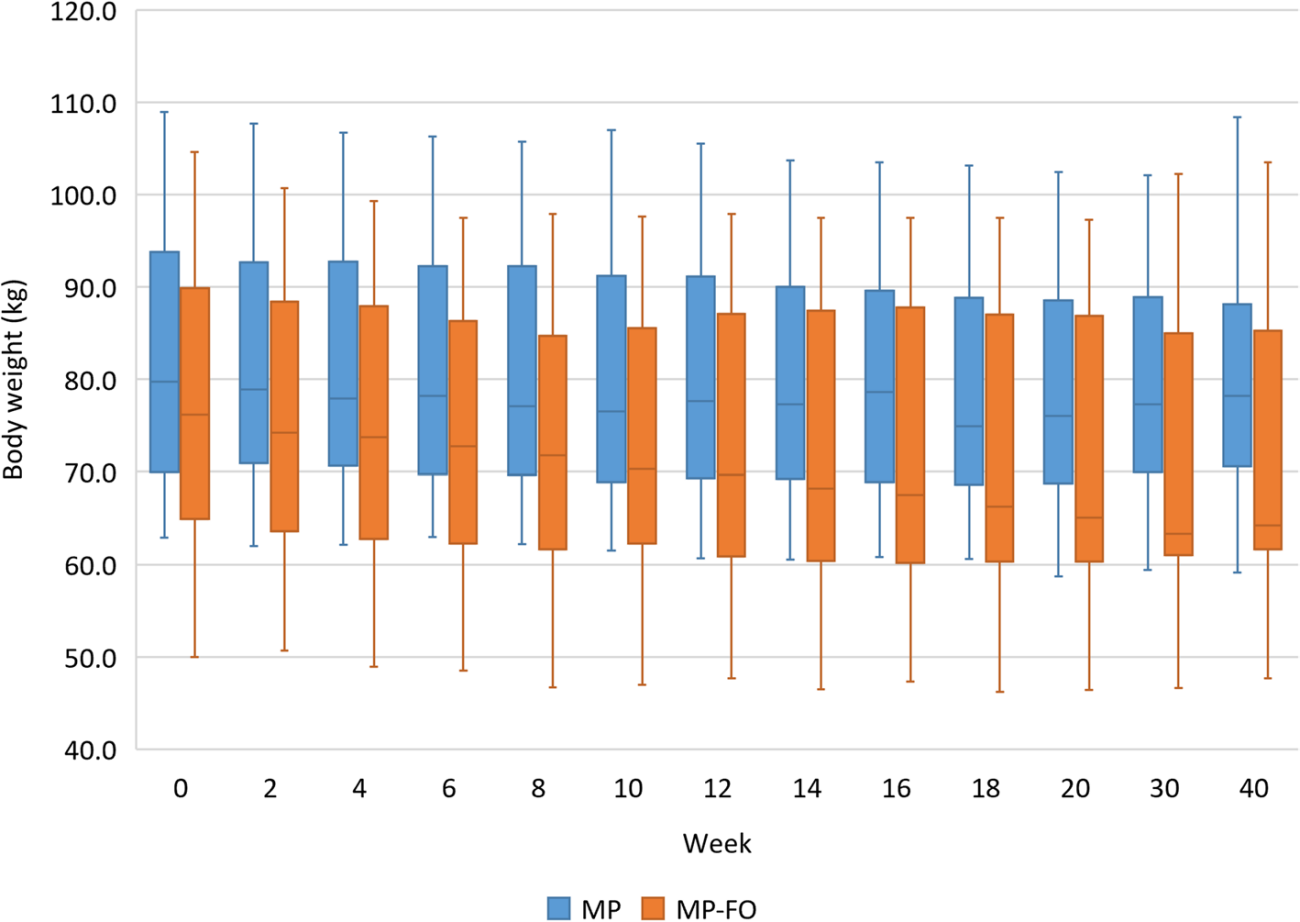
Body weight (kg) over the course of the MoKaRi study (13 times) in both study groups. Data at baseline (week 0), after weeks 2, 4, 6, 8, 10, 12, 14, 16, 18 and 20 as well as follow-up (week 30 and 40) are shown as median (25th, 75th percentile) according to the statistical test that was performed; times without a common letter are significantly different, *p* < 0.05; Abbreviations: MP, menu plan; MP-FO, menu plan plus fish oil

One of the strengths of the MoKaRi study design is the regular analysis of the study parameters at two-week intervals over the course of 20-week intervention period. In this way, the influence of variations in compliance during the 20 weeks of the intervention can be observed. In addition, the regular measurements can be used to determine the highest individual reduction in the primary outcome measure by implementing the concept (minimum values). A total of 51 subjects completed both the intervention and follow-up period of the MoKaRi study. Due to organizational challenges, complete data of all 13 times (every two weeks within the 20-week intervention period plus follow-ups at weeks 30 and 40) were only available of 12 participants in the MP group and 18 participants in the MP-FO group. These data sets show that body weight decreased within the first 8 weeks, and the reduction from baseline values was significant at weeks 10, 12, 14, 16, 18, and 20 in the MP group, (*p* < 0.05). In the MP-FO group, body weight dropped within the first 6 weeks and the differences from baseline were significant after 8, 10, 12, 14, 16, 18, and 20 weeks (*p* < 0.05). At weeks 14, 16, 18, and 20, a further reduction from week 6 was observed (*p* < 0.05). In both groups, body weight stayed at the reduced level after 10 and 20 weeks of follow-up. This reduction also differed from baseline (*p* < 0.05). No differences were found between both groups. The lowest average body weight was observed after 18 weeks in the MP group and after 30 weeks (after a 10-week follow-up) in the MP-FO group (Fig. 5).

**Fig. 6:**
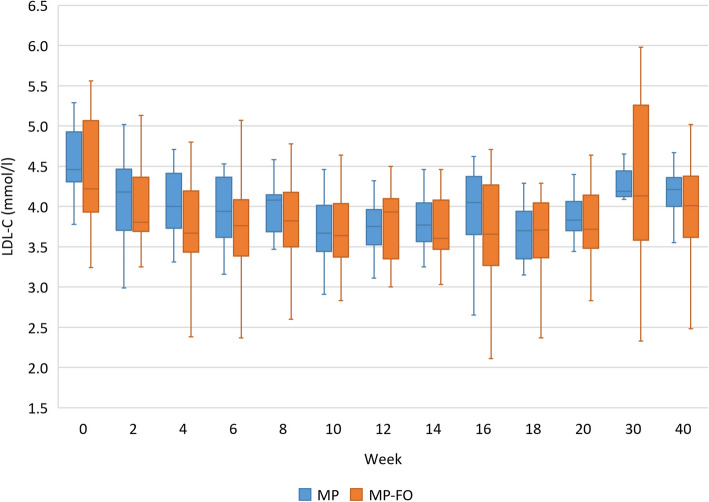
LDL-C (mmol/l) over the course of the MoKaRi study (13 times) in both study groups. Data at baseline (week 0), after weeks 2, 4, 6, 8, 10, 12, 14, 16, 18 and 20 as well as follow-up (week 30 and 40) are shown as median (25th, 75th percentile) according to the statistical test that was performed; times without a common letter are significantly different, *p* < 0.05; Abbreviations: LDL-C, low-density lipoprotein cholesterol; MP, menu plan; MP-FO, menu plan plus fish oil

To find out how quickly and how strong LDL-C was influenced by the MoKaRi concept, maybe depending on compliance, we monitored the changes over time at two-week intervals. Complete data of 11 participants in the MP group and 18 participants in the MP-FO group were available at 13 times (every 2 weeks within the 20-week intervention period and follow-up at weeks 30 and 40). In both groups, LDL-C levels decreased within the first 4 and 6 weeks, respectively, and the reduction from baseline always remained significant until the end of the intervention (*p* < 0.05). LDL-C increased within the follow-up (*p* < 0.05). No differences were observed between both groups. The lowest average LDL-C concentrations were measured after 10 weeks in the MP group and after 14 weeks in the MP-FO group (Fig. 6).

Next, we analysed the minimum body weight and LDL-C levels over the 20-week intervention period and looked at their differences between the two groups. The minimum values within the 20-wk intervention show the highest individual potential regarding the reduction of body weight and LDL-C by implementing the MoKaRi concept. In both groups, the baseline values for body weight (kg) and LDL-C (mmol/l) differed from the lowest values during the 20-week intervention (*p* < 0.001; Table 7). The reduction of body weight in the MP group (5%) differed that in the MP-FO group (9%; *p* < 0.05). Regarding LDL-C, the lower baseline and minimum concentrations in the MP-FO group differed significantly from the respective values in the MP group. However, the highest change in LDL-C from baseline was comparable between both groups (−25.9% vs. −26.6%; Table 7).

**Table 7:** Body weight (kg) and LDL-C (mmol/l) at baseline and the minimum values within the 20-week intervention period for both groups of the MoKaRi study

**Parameter**	**wk**	**MP group** **(*n* = 26)** Characteristics*	p Value within group	**MP-FO group** **(*n* = 25)** Characteristics*	p Value within group	p Value MP vs. MP-FO
Body weight (kg)	Baseline	80.6 (± 12.9) (62.2–108.9)	a	80.6 (± 16.8) (50.0–126.6)	a	0.990
	Min within 20 weeks	75.6 (± 12.0) (58.7–102.4)	b	73.4 (± 16.8) (46.2–124.1)	b	0.594
	Min Cfb	4.7 (2.2, 6.5) (0.8–12.0)		6.2 (3.8, 10.9) (1.0–16.2)		0.061
	Min Cfb^∆^	5.2 (2.8, 8.2) (1.1–14.2)		8.9 (5.5, 12.6) (1.5–23.5)		0.032*
LDL-C (mmol/l)	Baseline	4.7 (± 0.9) 4.8 (4.1, 5.2) (3.1–6.7)	a	4.0 (3.6, 4.9) (3.1–7.2)	a	0.046*
	Min within 20 weeks	3.5 (± 0.7) (2.2–4.6)	b	3.1 (± 0.6) 3.0 (2.8, 3.6) (2.1–4.1)	b	0.035*
	Min Cfb	1.1 (0.8, 1.6) (0.4–2.4)		1.0 (0.8, 1.4) (0.2–3.1)		0.678
	Min Cfb^∆^	25.9 (± 9.6) (10.0–45.0)		26.6 (± 11.4) (5.6–49.1)		0.807

^*^ Variables expressed as mean (± SD) (minimum–maximum) and/or as median (25th, 75th percentile) (minimum–maximum) depending on the statistical test that was performed; Cfb, change between baseline and the end of intervention (week 20); Cfb^∆^ percentage change between baseline and end of intervention (week 20); times without a common letter are significantly different, p < 0.001. Abbreviations; LDL-C, low-density lipoprotein cholesterol; MP, menu plan; MP-FO, menu plan plus fish oil; * p ≤ 0.05; ** p ≤ 0.01

The analysis of the individual data shows strong inter-individual differences in weight loss and LDL-C reduction. Some subjects in both groups achieved a weight loss of up to 12–16 kg, while others achieved a reduction of LDL-C up to 45 to 49% compared to the baseline values (Table 7).

### Effect of the MoKaRi diets on further cardiometabolic risk factors

Besides the data already presented, we wanted to investigate the effects on blood pressure and low-grade inflammation (Table 8). Systolic and diastolic blood pressure fell after within 10 weeks in both groups. In the MP group, there was a significant reduction also after 20 weeks. In the MP-FO group, systolic blood pressure tended to fall after 20 weeks; the reduction after the follow-up examination was significant (*p* < 0.05). Diastolic blood pressure in this group dropped after 10 and 20 weeks (*p* < 0.05). For pulse, the lower values at week 10 in the MP group differed from higher values measured at week 20 (*p* < 0.05; Table 6). High-sensitivity C-reactive protein (hs-CRP) decreased in the MP-FO group after 20 study weeks (*p* < 0.05). The reduction from baseline in the MP-FO group was higher than in the MP group (−43% vs. −19%; *p* < 0.05; Table 8), likely due to the higher intake of long-chain omega-3 fatty acids.

**Table 8:** Blood pressure and high-sensitivity CRP at baseline, after 10, 20, and 40 weeks of the MoKaRi study

**Parameter**	**wk**	**MP group** **(*n* = 26)** Characteristics*	p Value within group	**MP-FO group** **(*n* = 25)** Characteristics*	p Value within group	p Value MP vs. MP-FO
Systolic blood pressure (mmHg)	0	143.3 (± 17.9)	a	140.7 (± 18.3)	a	0.640
	10	135.1 (± 14.3)	b	132.2 (± 16.9)	b	0.545
	20	132.9 (± 17.2)	b	134.4 (± 15.5)	a,b	0.764
	40	134.1 (± 11.4)	a,b	133.0 (± 16.2)	b	0.800
	Cfb^∆^	−7.3 (± 10.6)		−5.0 (± 8.8)		0.418
Diastolic blood pressure (mmHg)	0	88.1 (± 9.6)	a	83.3 (± 10.4) 82.5 (77.8, 87.5)	a	0.130
	10	82.0 (± 9.5) 81.0 (78.0, 87.0)	b	76.0 (73.0, 82.3)	b	0.066
	20	80.8 (± 10.3)	b	79.5 (± 9.6) 78.0 (75.0, 84.0)	b,c	0.669
	40	85.7 (± 9.6)	a	83.9 (± 10.0) 80.5 (78.0, 88.8)	a	0.551
	Cfb^∆^	−8.4 (−14.0, 0.0)		−7.6 (−12.4, −1.4)		0.516
Pulse (beats per minute)	0	65.3 (± 7.9) 64.5 (59.5, 70.5)	a,b	66.0 (62.8, 77.3)	a	0.242
	10	63.0 (± 11.4) 61.5 (55.0, 68.8)	a	65.0 (59.8, 68.3)	a	0.478
	20	68.9 (± 10.7)	b	69.0 (± 7.7) 70.5 (64.3, 74.0)	a	0.960
	40	67.3 (± 8.2)	a,b	70.1 (± 10.7) 68.5 (63.5, 73.0)	a	0.359
	Cfb^∆^	3.3 (−3.5, 12.7)		−1.6 (−7.5, 3.5)		0.101
High-sensitivity CRP (mg/l)	0	2.4 (1.4, 3.5)	a	2.0 (1.1, 3.9)	a	0.523
	10	1.8 (1.2, 7.8)	a	1.7 (0.7, 3.4)	a,b	0.299
	20	2.0 (1.0, 3.4)	a	1.0 (0.6, 3.1)	b	0.154
	40	1.9 (1.4, 3.9)	a	1.7 (0.6, 3.3)	a,b	0.374
	Cfb^∆^	−19.4 (−36.8, 0.0)		−42.9 (−63.6, −12.5)		0.045*

^*^ Variables expressed as mean (± SD) and/or as median (25th, 75th percentile) depending on the statistical test that was performed; Cfb^∆^ percentage change between baseline and end of intervention (week 20); times without a common letter are significantly different, *p* < 0.05.

*Abbreviations*: *CRP *C-reactive protein, *DBP *diastolic blood pressure, *MP *menu plan, *MP-FO *menu plan plus fish oil, *SBP *systolic blood pressure; * p ≤ 0.05

### Effect of the MoKaRi diet on diabetes risk factors

To investigate the influence of the MoKaRi intervention on diabetes risk we analysed fasting glucose, insulin, and calculated HOMA-IR which have not worsened over the 20 and 40 weeks of the MoKaRi study (Table 9). Despite the relatively high carbohydrate intake recommended by the menu plans, the HbA1c was lower after 10, 20 and 40 weeks in both groups (*p* < 0.05). In the MP-FO group, we observed a larger reduction of HbA1c between weeks 10 and 20 which stabilised after 40 weeks (*p* < 0.05; Table 9).

**Table 9:** Diabetes risk factors at baseline, after 10, 20, and 40 weeks of the MoKaRi study

**Parameter**	**wk**	**MP group** **(*n* = 26)** Characteristics*	p Value within group	**MP-FO group** **(*n* = 25)** Characteristics*	p Value within group	p Value MP vs. MP-FO
Fasting glucose (mmol/l)	0	5.7 (5.5, 6.2)	a	6.0 (5.2, 6.4)	a,b	0.861
	10	6.1 (5.6, 6.6)	a,b	5.9 (5.4, 6.4)	a,b	0.584
	20	6.1 (5.7, 6.6)	b	5.9 (5.6, 6.7)	a	0.967
	40	5.7 (5.5, 6.1)	a,b	5.7 (5.4, 5.9)	b	0.577
	Cfb^∆^	4.3 (0.0, 8.8)		5.5 (0.0, 12.2)		0.843
Fasting insulin (mU/l)	0	8.5 (7.0, 13.3)	a	7.4 (5.5, 9.9)	a	0.187
	10	7.9 (6.2, 12.7)	a	6.4 (5.5, 8.0)	a,b	0.068
	20	9.5 (7.8, 12.3)	a	6.7 (5.5, 9.8)	a,b	0.061
	40	8.3 (6.1, 11.1)	a	6.3 (5.5, 7.2)	b	0.047*
	Cfb^∆^	4.1 (−12.7, 30.5)		−4.1 (−19.6, 15.6)		0.291
HOMA-IR	0	2.3 (1.6, 3.9)	a	1.9 (1.4, 2.9)	a	0.375
	10	1.9 (1.6, 3.7)	a	1.6 (1.3, 2.6)	a,b	0.108
	20	2.3 (2.0, 3.9)	a	1.8 (1.4, 2.9)	a	0.158
	40	2.0 (1.6, 3.4)	a	1.6 (1.3, 2.1)	b	0.070
	Cfb^∆^	10.1 (−12.3, 41.3)		1.0 (−15.2, 13.7)		0.318
HbA1c (%)	0	5.6 (5.3, 6.0)	a	5.6 (5.5, 5.8)	a	0.991
	10	5.5 (5.2, 5.7)	b	5.5 (5.4, 5.7)		0.757
	20	5.5 (5.4, 5.7)	b	5.4 (5.2, 5.7)	c	0.330
	40	5.5 (5.2, 5.7)	b	5.4 (5.1, 5.6)	c	0.466
	Cfb^∆^	−1.8 (−3.5, 0.0)		−3.6 (−5.5, −1.8)		0.041*

^*^ Variables expressed as mean (± SD) and/or as median (25th, 75th percentile) depending on the statistical test that was performed; Cfb^∆^ percentage change between baseline and end of intervention (week 20); times without a common letter are significantly different, *p* < 0.05

*Abbreviations*: *HbA1c *glycated hemoglobin A_1c_, *HOMA-IR *homeostatic model assessment for insulin resistance, *MP *menu plan, *MP-FO *menu plan plus fish oil; * p ≤ 0.05

Except for the reduction of the quick value in the MP group (*p* < 0.05), the clotting markers and blood count did not change significantly throughout the study or between both groups (Table S2).

## Discussion

### Effect of the MoKaRi diets on nutrient status

In addition to the optimized intake of energy and major nutrients such as carbohydrates, protein, dietary fibers and fat amount and quality, the 140 individualised menu plans which were developed for the MoKaRi study covered at least the daily requirement of vitamins (except for vitamin D, which is partially covered by UVB radiation from sunlight), minerals and trace elements (except for selenium, as no data on selenium concentration in food is available from our software tool (PRODI® version 6.4, Nutri-Science, Stuttgart, Germany).

The analyzed nutrients in human plasma or serum and 24-h urine reflecting nutrient status show only marginal changes in response to the 20-week implementation of the MoKaRi menu plans. In detail, vitamin B_6_, C, and beta-carotene increased, but this was only significant in the MP-FO group. Vitamin D increased in both groups, which is mainly attributed to the change in sunlight exposition (UVB radiation) from February (baseline) to June/July (end of the 20-week intervention). On the other hand, holo-TC, iron, beta-cryptoxanthin, alpha- and gamma-tocopherol decreased in the MP-FO group, and calcium, ferritin, and iodine decreased in both groups (*p* < 0.05). The observed changes could be due to i) the reduction of daily meat and sausage consumption, which was limited to 2 to 4 times per week, ii) the reduction of table salt, which in Germany is predominantly fortified with iodine, and iii) the recommended daily consumption of plant-based foods, e.g. vegetables, legumes, berries and foods rich in whole grain fibers, which could limit the bioavailability of minerals and trace elements.

Based on the dietary records over seven days before baseline (run-in period), the intake of some nutrients with unfavourable physiological effects, such as SFA, cholesterol, sodium, chloride, and phosphorus, was too high, and the intake of valuable nutrients, such as MUFA, PUFA, vitamin A, calcium, potassium, and iodine did not meet the recommendations of the German Nutrition Society (**DGE**). The analysis of the nutrient status does not cover all these identified critical nutrients, but our data show that the daily intake of required amounts of, e.g. vitamin B_6_ and vitamin C by the menu plans caused an increase in their concentrations. The expected improvement in the supply of vitamin A, calcium, potassium, and iodine through the daily implementation of the menu plans, which theoretically deliver these nutrients according to recommendations, cannot be mapped. This may be due to a discrepancy between the vitamins, minerals and trace elements consumed by the MoKaRi participants and the nutrient data stored in the database used for the calculation (PRODI® Version 6.4, Nutri-Science, Stuttgart, Germany) as micronutrient content of food depends on certain factors such as variety, growing region, processing, etc., which are not taken into account in the entries in the database. In addition, due to the complex and cost-intensive determination methods for each individual micronutrient, the micronutrient information is incomplete for all foods.

The observed discrepancy in dietary fat quality between dietary guidelines and actual intake was reduced by the menu plan-based intervention, as total SFA decreased and MUFA increased significantly in the MP group. The recommendation of regular intake of linseed oil and sea fish as sources of n-3 PUFA by the menu plans resulted in a moderate increase of the n-3 index in this group. Due to additional fish oil supplementation in the MP-FO group, the n-3 index has more than doubled. The reached n-3 index in the target range of 8–11% is associated with lower total mortality and fewer major adverse cardiac and other cardiovascular events [26].

In addition, the total n-6 PUFA concentrations decreased in the MP-FO group, leading to a sharp decline in the n-6/n-3 PUFA ratio, especially the AA/EPA and AA/DHA ratios. The incorporation of EPA and DHA in membrane phospholipids at the expense of AA influences the production of lipid mediators, which are generated from membrane phospholipids [27]. Numerous studies in healthy human volunteers and patients with chronic inflammatory diseases have described decreased production of 2-series PGs and 4 series-LTs by inflammatory cells following the use of marine n-3 fatty acid supplements for a period of weeks to months [27]. In addition, EPA and DHA are precursors of resolvins, protectins and maresins with anti-inflammatory and inflammation-resolving effects, which have been extensively shown in cell culture and animal models of inflammation but also in patients with chronic inflammatory diseases [27–30].

To sum up, implementing the MoKaRi concept leads to an improvement in the intake of macronutrients such as carbohydrates, dietary fibers and, in particular, fat quality. The intake of micronutrients could only be partially improved by menu plans that have theoretically specified quantities of vitamins, minerals, and trace elements to cover requirements.

### Effect of the MoKaRi diets on cardiometabolic risk factors

The implementation of the MoKaRi concept resulted in an effective reduction of cardiometabolic risk factors such as body weight, TC, LDL-C, TG, HbA1c, and blood pressure.

The daily menu plans limited the intake of SFA to 7 En% by banning fast foods and reducing the daily consumption of sausage, pork, high-fat dairy products, palm oil, and coconut oil. Dietary SFA decrease hepatic low-density lipoprotein (LDL) receptor, thus decreasing the clearance of circulating LDL [30–32]. Chiu et al. [33] confirmed the impact of SFA in a parallel-designed study with 53 individuals with atherogenic dyslipidemia as characterized by a preponderance of small LDL particles (LDL phenotype B). A diet low vs. high in SFA resulted in a decrease of small LDL particles by about 21% (CI [−32.8 to −6.7%]) vs. an increase of 6% (CI [−10.3 to 25.6%]); *p* = 0.02]. A comparable effect was seen for total LDL (3.6 mmol, CI [−3.2 to 11.0] vs. −7.9 mmol, CI [ −13.9 to −1.5]; *p* = 0.03). This data is consistent with our results and points out that the SFA restriction by the menu plans contributes to the observed beneficial effect on TC, LDL-C, and sdLDL-C.

In addition, the daily consumption of MUFA and PUFA sources, e.g. olive oil, canola oil, avocados, and various nuts and seeds was recommended by the menu plans and further contributes to lower TC and LDL-C by increasing hepatic LDL receptor, that in turn increases the clearance of LDL from the circulation [32]. PUFA are a preferred substrate for acyl-CoA:cholesterol acyltransferase (ACAT) resulting in increased cholesteryl ester formation and decreased free cholesterol in the liver [30]. This causes an up-regulation of the LDL receptor. In addition, PUFA also increased membrane fluidity, leading to an increase in the ability of the LDL receptor to bind LDL [31].

Replacing SFA with MUFA, particularly PUFA, in the diet lowers LDL-C without affecting HDL-C and TG [32, 34]. Supplementation of EPA and DHA by oils from marine sources has no substantial effect on LDL-C but dose-dependently lowers TG concentrations [9, 10], which is concurrent with our data.

The impact of dietary cholesterol on LDL-C is modest and varies strongly, but approximately 15–25% of individuals are hyper-responders and react with a more robust increase of LDL-C [32]. The increase in LDL-C by dietary cholesterol is carried due to a decrease in hepatic LDL receptors, leading to a decrease in the clearance of LDL from the circulation [35]. In addition, the decrease in LDL receptor could increase the conversion of intermediate density lipoproteins to LDL rather than clearance by the liver (i.e., LDL production is enhanced) [32]. To address this relation, the consumption of egg yolks, shrimp, beef, pork, poultry, cheese, and butter was limited by the menu plans to decrease the regular intake of dietary cholesterol to at least 300 mg daily.

The design of the MoKaRi study allows the evaluation of an additional benefit of fish oil supplementation on cardiometabolic risk factors. Our data show a more substantial effect on the reduction of body weight and BMI (−5% vs. −8%), TG (−14% vs. −28%), hs-CRP (−19% vs. −43%), and HbA1c (−2% vs. −4%) in the MP-FO group (*p* < 0.05), indicating a larger effect of the MoKaRi intervention on cardiometabolic risk factors due to the additional fish oil supplementation. In their meta-analysis, Gao et al. [36] confirmed the TG-lowering effect by fish oil supplementation but found no effect on glucose control. The data on hs-CRP are in line with the meta-analysis by Taha et al. [37]. A systematic review and meta-analysis by Bender et al. [38] found evidence that participants taking fish or fish oil lost 0.59 kg (CI [−0.96 to −0.21]) more body weight than controls. In addition, treatment groups lost 0.24 kg/m^2^ more BMI points than controls and 0.5% more body fat than controls.

Besides, we observed a larger reduction of apolipoprotein A1 (−15% vs. −20%) and a smaller reduction of apolipoprotein B (−15% vs. −6%) in the MP-FO group (*p* < 0.05). These effects slightly diminish the benefits of combining menu plans and fish oil supplementation.

The effects of n-3 PUFA on cardiovascular events or mortality are contradictory. While studies with low dosages (approx. 1 g/d EPA + DHA) failed to lower the incidence of major cardiovascular events, the *Reduction of Cardiovascular Events with Icosapent Ethyl-Intervention Trial* (REDUCE-IT) study using a higher dose (4 g/d of a highly purified EPA ethylester) found a remarkable, statistically significant reduction in CVD events [39–41]. A recent meta-analysis based on data on EPA and DHA status measured by gas chromatography demonstrates the benefit of n-3 PUFA on cardiovascular risk. The authors showed that a higher relative proportion of long-chain n-3 PUFA (EPA, DPA, DHA) was associated with 18% lower odds of cardiovascular death [42]. Long-chain n-3 PUFA concentrations in the top quartile were related to 51% lower odds of cardiovascular death and 63% lower odds of sudden cardiac death compared to the lowest quartile.

Furthermore, Gao et al. [43] demonstrated the benefit of EPA + DHA on coronary atherosclerosis in their meta-analysis. n-3 PUFA were associated with a reduction of the atherosclerotic plaque volume (standardized mean difference (SMD) −0.18, CI [−0.31 to −0.05]), and they reduce the loss of the diameter of the narrowest segments of coronary arteries in patients with CHD (SMD 0.29, CI [0.05—0.53]).

Besides the optimization of fat intake and fat quality, the menu plans also improve carbohydrate quality by increasing the intake of high-quality carbohydrates from berries, legumes, vegetables, and whole grains, and limiting the intake of low-quality carbohydrates from refined grains and added sugar. This improvement in carbohydrate quality caused a reduction of HbA1 in both groups. This may have contributed to the observed reduction in TC, LDL-C and TG, as a link between blood glucose concentrations and serum lipids has been demonstrated [32, 44]. Complementary, Aeberli et al. [45] showed that a regular low to moderate consumption of fructose or sucrose (40–80 g/d) in healthy young men results in a marked reduction of LDL particle size and an increase of fasting glucose and hs-CRP (*p* < 0.05).

Dietary fiber is found primarily in berries, vegetables, whole and unrefined grains, nuts, seeds, beans, and legumes [32]. It is estimated that for each additional gram of soluble fiber in the diet, TC and LDL-C concentrations decrease by −0.028 mmol/L and −0.029 mmol/L, respectively [16]. Trautwein and McKay [46] concluded that an intake of 4–10 g/d of different types of soluble fiber, such as beta-glucan from oats and barley, psyllium, and glucomannan is required to achieve a 5–10% reduction in LDL-C without substantially affecting HDL-C and TG concentrations.

In summary, the current literature shows that dietary changes based on replacing SFA with MUFA and PUFA and increasing consumption of plant foods such as vegetables, berries, legumes, nuts and whole grains rich in phytosterols and fiber can reduce plasma cholesterol concentrations by 10–30% in individuals with non-familial hypercholesterolemia [46, 47]. The recommendations mentioned above are included in the MoKaRi concept and led to an average reduction in LDL-C of 14–16% within the 20-wk intervention period.

By taking regular blood samples every 2 weeks we were able to track the progress of blood lipids over the whole intervention period. This analysis shows a clear relation between reduced LDL-C concentrations and daily adherence to the menu plans. It should be noted that at the end of the intervention phase (June/July), the reduction of TC and LDL-C had slightly declined. We speculate that this effect could be due to the start of the barbecue season in Thuringia. To demonstrate the maximum effect of the MoKaRi approach on the primary outcome measure, we compared the minimum LDL-C concentrations during the 20-week intervention with the baseline values and found an average reduction in LDL-C of 24–26%. Some subjects even achieved a reduction in LDL-C by almost 50%.

Overall, we assume that the reduction in TC and LDL-C due to the MoKaRi concept is multifactorial. We hypothesize effects on LDL receptor activity due to (i) the limitation in fat intake, (ii) the improvement in fat quality, and (iii) the limitation of cholesterol intake. In addition, the change in bile acid metabolism due to the stimulated intake of dietary fiber will have contributed to the observed reduction in blood lipids [17]. Our data point out that the observed effects of blood lipids seem to be independent from the reduction of body weight as we found no correlation between the reduction in body weight and the changes of LDL-C (r −0.031; *p* = 0.751).

SCORE2 is a validated instrument to predict 10-year risk of first-onset CVD in European populations [48]. The implementation of the MoKaRi concept resulted in a reduction from 10.2 ± 6.6% to 8.3 ± 6.0% in the MP group and from 7.6 ± 4.6% to 7.1 ± 5.0% in the MP-FO group. We assume that this score does not reflect total potential of risk reduction, as LDL-C and diastolic blood pressure are not directly taken into account, and the protective effect of HDL-C is increasingly being discussed [49].

Besides the improvement in TC and LDL-C, we also observed a marked reduction in further cardiometabolic risk factors, such as body weight, BMI, waist circumferences, apolipoprotein B, HbA1c, and diastolic blood pressure due to the implementation of the menu plans in both groups.

With regard to weight reduction, the following aspect should also be taken into account. Menu plans provided a needs-based amount of energy, as recommended by the German Nutrition Society for adults. Accordingly, only overweight participants lost weight, while normal-weight subjects did not lose any weight. The reduction in diastolic blood pressure could be explained by the fact that the menu plans set salt consumption at approx. 6 g/day.

Overall, the MoKaRi study proves the benefit of our dietary intervention on cardiometabolic risk factors. Comparable effects due to dietary advice are described by Kahleova et al. [50]. In addition, a systematic review based on 153 studies (one randomized controlled trial and 152 prospective cohort studies) provides strong evidence for an association of dietary patterns fitting the characteristics of the MoKaRi concept (encouraged consumption of vegetables, fruits, legumes, nuts, whole grains, unsaturated vegetable oils, and fish, lean meat or poultry if meat was included and limited intake of red and processed meat, high-fat dairy, and refined carbohydrates or sweets) with a decreased risk of all-cause mortality in adults and older adults [51]. This underlines the importance of the long-term implementation of a healthy diet, e.g. following the evidence-based and validated criteria of the MoKaRi concept.

## Conclusion

The MoKaRi study provides essential insights into the relationship between diet, lifestyle and cardiometabolic risk factors. The implementation of the MoKaRi concept, which is based on diet-based information and practical tools, ready-to-use in day-by-day routine (e.g. the 140 menu plans following the MoKaRi criteria for optimized energy and nutrient intake), caused the improvement of TC and LDL-C, as well as an apparent improvement of further cardiometabolic risk factors, such as body weight, BMI, waist circumferences, apolipoprotein B, HbA1c, and diastolic blood pressure. Over the study time, a maximum reduction in LDL-C concentration by an average 26–27% was achieved in both groups, individual participants of achieve a reduction of up to 45–49%. Our data highlights the additional benefit of the combination between menu plans and fish oil supplementation, which resulted in more substantial effects on body weight, BMI, TG, HbA1c and hs-CRP.

The reduction in TC and LDL-C is due to limiting SFA intake to below 7 En% and increasing fiber intake by at least 40 g/d through the menu plans. The positive effects on other study parameters are due to the holistic approach, as the entire diet was modulated. To sum up, the validated MoKaRi concept counteracts high-normal blood pressure, impaired fasting glucose, and overweight which are strong predictors of hypertension, type 2 diabetes, and obesity. Since the lower the LDL-C value, the greater the cardiovascular protection [18, 21], the LDL-lowering effect resulting from the implementation of the MoKaRi menu plans should be particularly emphasized.

## Strengths and limitations

The scientific evidence-based and practically oriented MoKaRi concept and the comprehensive health status assessment focusing on cardiometabolic risk factors are the strengths of the MoKaRi study. In addition, regular blood sample taking every 2 weeks throughout the study enables the evaluation of the progress of study parameters. Besides the close personal supervision by the study team various strategies to increase motivation and compliance to follow the advice given by the menu plans are further strengths.

The main limitation of the MoKaRi study is the lack of a control group without dietary recommendations following their usual dietary habits.

In addition, the gender ratio is not balanced, as most of our participants were women. This might be due the fact that women have a greater interest in the topics of nutrition and health. In addition, women are usually responsible for buying food, preparing meals, etc. and are therefore more likely to deal with these issues.

Further, a diet based on MoKaRi menu plans enables theoretically precise control of the subjects' nutrient intake, but the implementation of deviations from the menu plans in the daily routine depends heavily on the motivation of the participants. This could be a challenge as the menu plans are based on the preparation of 3–5 meals per day, which is very time consuming. In addition, the purchase of ingredients causes costs that can be higher than without the concept.

Apart from analysing the fatty acid distribution in the plasma and erythrocyte lipids and directly querying the compliance, we unfortunately have no other means of checking the daily implementation of the menu plans.

The menu plans were prepared with the nutrition software PRODI® (version 6.4, Nutri-Science, Stuttgart, Germany), which uses the data set of the ‘Bundeslebensmittelschlüssel’ to calculate nutrient profiles for all foods recommended by the plans. We have to consider discrepancies between the nutrients consumed by the participants and the nutrient data stored in the database used for the calculation. This is particularly relevant for the micronutrient content of foods, which depends on certain factors such as variety, growing region, processing, etc. This could not be considered in the database. In addition, micronutrient information is not complete for all foods due to the complex and cost-intensive determination methods for each micronutrient.

## Supplementary Information

Additional file 1: Table S1. Biochemical methods [52–54].

## Data Availability

No datasets were generated or analysed during the current study.
