# Supplementary material for: Reduction of cardiovascular risk factors by the diet – Evaluation of the MoKaRi concept by a parallel-designed randomized study
Source: Lipids Health Dis. 2025 Mar 8;24:88. doi: 10.1186/s12944-025-02500-1 (PMC11889781; doi:10.1186/s12944-025-02500-1)
Supplement: Supplementary file 1 — Additional file 1: Table S1. Biochemical methods [52–54]. [file 12944_2025_2500_MOESM1_ESM.docx]

**Supplemental materials**

**Table S1.** Biochemical methods.

| **Parameter** | **Instrument** | **Reference range** |
| --- | --- | --- |
| Plasma, serum | | |
| Total cholesterol (mmol/l)* | Abbott Architect CI 16200 analyzer  (Abbott, Wiesbaden, Germany) | < 5.2 |
| HDL cholesterol (mmol/l)* | Abbott Architect CI 16200 analyzer  (Abbott, Wiesbaden, Germany) | > 1.03 |
| LDL cholesterol (mmol/l)* | Abbott Architect CI 16200 analyzer  (Abbott, Wiesbaden, Germany) | < 3.35 |
| Triglycerides (mmol/l)* | Abbott Architect CI 16200 analyzer  (Abbott, Wiesbaden, Germany) | < 1.7 |
| Lipoprotein a (mg/l)* | Abbott Architect CI 16200 analyzer  (Abbott, Wiesbaden, Germany) | 0.85 – 1.15 |
| Apolipoprotein A1 (g/l)^◊^ | COBAS INTEGRA 400 plus System  (Roche, Mannheim, Germany) | women: 1.08 – 2.25  men: 1.04 – 2.02 |
| Apolipoprotein B (g/l)^◊^ | COBAS INTEGRA 400 plus System  (Roche, Mannheim, Germany) | women: 0.60 – 1.17  men: 0.66 – 1.33 |
| MDA-LDL (U/l)^◊^ | Manual MDA-LDL ELISA  (ImmBioMed, Pfungstadt, Germany) | No information available |
| Homocystein (µmol/l)* | HPLC (Shimadzu, Kyoto, Japan) | 5 – 15 |
| High-sensitive troponin (pg/ml)* | Abbott Architect i2000 (Abbott, Wiesbaden, Germany) |  |
| Retinol (µmol/l)# | HPLC (Shimadzu, Kyoto, Japan) | not applicable |
| alpha-tocopherol (µmol/l)# | HPLC (Shimadzu, Kyoto, Japan) | not applicable |
| gamma-tocopherol (µmol/l)# | HPLC (Shimadzu, Kyoto, Japan) | not applicable |
| Carotenoids (µmol/l) (Lutein, zeaxanthin, beta-cryptoxanthin, lycopene, alpha-carotene, beta-cartotene)# | HPLC (Shimadzu, Kyoto, Japan) | not applicable |
| Malondialdehyde (µmol/l)# | HPLC (Shimadzu, Kyoto, Japan) | not applicable |
| High-sensitivity CRP (mg/l)* | Abbott Architect CI 16200 analyzer  (Abbott, Wiesbaden, Germany) | ≤ 0.3 |
| Glucose (mmol/l)* | Abbott Architect CI 16200 analyzer  (Abbott, Wiesbaden, Germany) | 18 – 60 y: 4.1 – 5.9  60 – 90 y: 4.6 – 6.4 |
| Insulin (mU/l)* | Abbott Architect CI 16200 analyzer  (Abbott, Wiesbaden, Germany) | 3 – 25 |
| HbA1c (%)* | Tosoh HLC-723G11  (Sysmex, Norderstedt, Germany) | 4.5 – 6.1 |
| Biotin (ng/l)^◊^ | IDK Biotin ELISA (Immundiagnostik, Bensheim, Germany) | > 250 |

| **Parameter** | **Instrument** | **Reference range** |
| --- | --- | --- |
| Plasma, serum | | |
| Folic acid (ng/ml)* | Abbott Architect CI 16200 analyzer  (Abbott, Wiesbaden, Germany) | 3.9 – 26.8** |
| Vitamin B_1_ (nmol/l)* | HPLC (Shimadzu, Kyoto, Japan) | 47 – 1412 |
| Vitamin B_2_ (µg/l)* | HPLC (Shimadzu, Kyoto, Japan) | 180 – 295 |
| Vitamin B_6_ (nmol/l)* | HPLC (Shimadzu, Kyoto, Japan) | 14.6 – 72.8 |
| Vitamin B_12_ (pmol/l)* | Abbott Architect CI 16200 analyzer  (Abbott, Wiesbaden, Germany) | 197 – 7712 |
| Holo-Transcobalamine (pmol/l)* | Abbott Architect CI 16200 analyzer  (Abbott, Wiesbaden, Germany) | > 37.52 |
| Vitamin C (mg/l)* | HPLC (Shimadzu, Kyoto, Japan) | 4 – 15 |
| Vitamin A (µmol/l)* | HPLC (Shimadzu, Kyoto, Japan) | 1.46 – 2.84** |
| Vitamin D (nmol/l)* | Abbott Architect CI 16200 analyzer  (Abbott, Wiesbaden, Germany) | > 75 |
| Vitamin E (µmol/l)* | HPLC (Shimadzu, Kyoto, Japan) | 11.6 – 46.4 |
| Calcium (mmol/l)* | Abbott Architect CI 16200 analyzer  (Abbott, Wiesbaden, Germany) | 2.15 – 2.50 |
| Potassium (mmol/l)* | Abbott Architect CI 16200 analyzer  (Abbott, Wiesbaden, Germany) | 3.4 – 4.5 |
| Iron (µmol/l)* | Abbott Architect CI 16200 analyzer  (Abbott, Wiesbaden, Germany) | women: 9.0 – 30.4  men: 11.6 – 31.1 |
| Ferritin (µg/l)* | Abbott Architect CI 16200 analyzer  (Abbott, Wiesbaden, Germany) | women: 13 – 150  men: 30 – 400 |
| Transferrin (g/l)* | Abbott Architect CI 16200 analyzer  (Abbott, Wiesbaden, Germany) | 2.0 – 3.6 |
| Iodine (µg/l)^◊^ | ICP/MS | 45 - 71 |
| aPTT (s)* | ACL TOP 750 CTS  (Fa Werfen, Munich, Germany) | 25.1 – 36.5 |
| Fibrinogen (g/l)* | ACL TOP 750 CTS  (Fa Werfen, Munich, Germany) | 2.8 – 4.7 |
| Quick (%)* | ACL TOP 750 CTS  (Fa Werfen, Munich, Germany) | 70 – 130 |
| INR* | ACL TOP 750 CTS  (Fa Werfen, Munich, Germany) | 0.85 – 1.15*** |
| Basophilic granulocytes (Gpt/l)* | XN 9100  (Sysmex, Norderstedt, Germany) | < 0.2 |
| Eosinophilic granulocytes (Gpt/l)* | XN 9100  (Sysmex, Norderstedt, Germany) | < 0.5 |
| Neutrophilic granulocytes (Gpt/l)* | XN 9100  (Sysmex, Norderstedt, Germany) | 1.8 – 7.7 |
| **Parameter** | **Instrument** | **Reference range** |
| Lymphocytes (Gpt/l)* | XN 9100  (Sysmex, Norderstedt, Germany) | 1 – 4.8 |
| Monocytes (Gpt/l)* | XN 9100  (Sysmex, Norderstedt, Germany) | < 0.8 |
| Leucocytes (Gpt/l)* | XN 1000  (Sysmex, Norderstedt, Germany) | 4.4 – 11.3 |
| Thrombocytes (Gpt/l)* | XN 1000  (Sysmex, Norderstedt, Germany) | 150 – 360 |
| Erythrocytes (Tpt/l)* | XN 1000  (Sysmex, Norderstedt, Germany) | women: 4.1 – 5.1  men: 4.5 – 5.9 |
| Hemoglobin (mmol/l)* | XN 9100  (Sysmex, Norderstedt, Germany) | 7.6 – 9.5 |
| MCV (fl)* | XN 9100  (Sysmex, Norderstedt, Germany) | 80 – 96 |
| 24h urine | | |
| Creatinine 24h urine (mmol/24h)* | Abbott Architect CI 16200 analyzer  (Abbott, Wiesbaden, Germany) | 8.0 – 26.5 |
| Methylmalonic acid 24 h urine (mmol/24h)* | LC-MS/MS  (Chromsystems Instruments & Chemicals GmbH, Munich, Germany) | < 2 |
| Magnesium 24h urine (mmol/24h)* | Abbott Architect CI 16200 analyzer  (Abbott, Wiesbaden, Germany) | No information available |
| Sodium 24h urine (mmol/24h)* | Abbott Architect CI 16200 analyzer  (Abbott, Wiesbaden, Germany) | 94 – 222 |
| Selenium 24h urine (µmol/24h)* | AAS ZEEnit 60  (Analytik Jena AG, Jena, Germany) | No information available |
| Zinc 24h urine (µmol/24h)* | AAS 5 FL1  (Analytik Jena AG, Jena, Germany) | No information available |
| Erythrocytes, plasma | | |
| Fatty acids (% FAME)^$^ | GC-17V3  (Shimadzu, Duisburg, Germany) | No information available |

* Measured by Institute of Clinical Chemistry and Laboratory Diagnostics, University Hospital Jena, Germany; accredited reference laboratory (D-PL-13144-03-00, valid until 05.06.2023).

◊ Measured by Dianovis GmbH, Greiz, Germany.

$ Measured by Institute of Nutritional Sciences, Friedrich Schiller University, Jena, Germany as previously described by Dittrich et al. [22].

# Measured by Department of Molecular Toxicology, German Institute of Human Nutrition Potsdam-Rehbruecke (DIfE) as previously described by Weber et al. [52].

** [53]

*** [54]

Abbreviations: AAS atomic absorption spectroscopy; aPTT, activated partial thromboplastin time; CRP, c-reactive protein; FAME, fatty acid methyl ester; GC, gas chromatography; HbA1c, glycated hemoglobin A1c; HDL, low-density lipoprotein; INR, international normalized ratio; LC-MS/MS, liquid chromatography-mass spectrometry; LDL, low-density lipoprotein; MCV, mean corpuscular volume; MDA-LDL, malondialdehyd-modified-low-density lipoprotein.

**Table S2:** Blood count and clotting parameters at baseline, after 10, 20, and 40 weeks of the MoKaRi study.

| **Parameter** | **wk** | **MP group  (n = 26)**  **Characteristics*** | p Value within group | **MP-FO group  (n = 25)**  **Characteristics*** | p Value within group | p Value MP vs. MP-FO |
| --- | --- | --- | --- | --- | --- | --- |
| aPTT  (s) | 0 | 31.00 (± 3.17) | a | 30.80 (± 2.94) | a | n.s. |
|  | 10 | 31.59 (± 3.10) | a | 31.56 (± 2.48) | a | n.s. |
|  | 20 | 31.41 (± 2.91) | a | 30.83 (± 2.20) | a | n.s. |
|  | 40 | 31.09 (± 2.66) | a | 30.81 (± 2.60) | a | n.s. |
|  | Cfb^∆^ | 0.33 (-5.67, 7.14) |  | 0.30 (-4.68, 4.10) |  | n.s. |
| Fibrinogen  (g/l) | 0 | 2.55 (± 0.29) | a | 2.57 (± 0.40)  2.50 (2.35, 2.65) | a,b | n.s. |
|  | 10 | 2.74 (± 0.49)  2.65 (2.40, 3.00) | a,b | 2.40 (2.20, 2.80) | a | 0.045 |
|  | 20 | 2.60 (± 0.30) | a | 2.47 (± 0.35)  2.50 (2.15, 2.75) | a | n.s. |
|  | 40 | 2.85 (± 0.45) | b | 2.75 (± 0.38)  2.70 (2.50, 3.00) | b | n.s. |
|  | Cfb^∆^ | 3.30 (± 12.11) |  | -2.84 (± 12.25) |  | n.s. |
| Quick  (%) | 0 | 105.00 (96.00, 109.25) | a | 107.00 (94.00, 112.00) | a | n.s. |
|  | 10 | 102.50 (93.00, 107.25) | a,b | 104.00 (98.50, 110.00) | a | n.s. |
|  | 20 | 97.00 (89.00, 105.25) | b | 103.00 (94.00, 110.00) | a | n.s. |
|  | 40 | 104.00 (96.75, 108.00) | a | 103.00 (98.00, 110.00) | a | n.s. |
|  | Cfb^∆^ | -4.09 (-13.20, 0.98) |  | -2.13 (-6.25, 2.83) |  | n.s. |
| INR | 0 | 1.00 (0.98, 1.00) | a | 1.00 (0.90, 1.00) | a | n.s. |
|  | 10 | 1.00 (1.00, 1.10) | a | 1.00 (0.90, 1.00) | a | n.s. |
|  | 20 | 1.00 (1.00, 1.10) | a | 1.00 (0.95, 1.05) | a | n.s. |
|  | 40 | 1.00 (1.00, 1.00) | a | 1.00 (0.95, 1.00) | a | n.s. |
|  | Cfb^∆^ | 5.00 (0.00, 11.11) |  | 0.00 (0.00, 10.00) |  | n.s. |
| Basophilic granulocytes  (Gpt/l) | 0 | 0.03 (0.02, 0.04) | a | 0.03 (0.02, 0.05) | a | n.s. |
|  | 10 | 0.03 (0.02, 0.04) | a | 0.03 (0.02, 0.04) | a | n.s. |
|  | 20 | 0.05 (± 0.02)  0.05 (0.04, 0.06) | b | 0.06 (± 0.02)  0.05 (0.04, 0.07) | b | n.s. |
|  | 40 | 0.04 (0.04, 0.05) | b | 0.05 (0.03, 0.06) | b | n.s. |
|  | Cfb^∆^ | 50.00 (33.33, 93.75) |  | 50.00 (16.67, 100.00) |  | n.s. |
| Eosinophilic granulocytes  (Gpt/l) | 0 | 0.17 (0.12, 0.19) | a | 0.12 (0.10, 0.24) | a | n.s. |
|  | 10 | 0.17 (0.14, 0.22) | a | 0.15 (0.10, 0.26) | a | n.s. |
|  | 20 | 0.20 (± 0.08)  0.17 (0.15, 0.27) | a | 0.19 (± 0.11)  0.15 (0.11, 0.23) | a | n.s. |
|  | 40 | 0.20 (± 0.08)  0.18 (0.15, 0.22) | a | 0.20 (± 0.09)  0.19 (0.14, 0.24) | a | n.s. |
|  | Cfb^∆^ | 9.01 (± 30.67) |  | 9.85 (± 46.6) |  | n.s. |
| Neutrophilic granulocytes  (Gpt/l) | 0 | 4.0 (3.1, 4.3) | a | 3.6 (3.0, 4.6) | a | n.s. |
|  | 10 | 3.4 (3.0, 3.8) | a | 3.1 (2.6, 4.0) | a | n.s. |
|  | 20 | 3.1 (2.9, 3.8) | a | 3.1 (2.7, 4.1) | a | n.s. |
|  | 40 | 3.4 (± 0.8)  3.4 (2.9, 3.7) | a | 3.4 (± 1.3)  3.1 (2.5, 4.3) | a | n.s. |
|  | Cfb^∆^ | -3.0 (-14.9, 4.7) |  | -10.9 (-33.1, 7.4) |  | n.s. |

| Lymphocytes  (Gpt/l) | 0 | 1.9 (± 0.4)  1.9 (1.7, 2.1) | a | 1.9 (± 0.5) | a | n.s. |
| --- | --- | --- | --- | --- | --- | --- |
|  | 10 | 2.0 (± 0.6)  1.8 (1.8, 2.3) | a | 1.9 (± 0.6) | a | n.s. |
|  | 20 | 2.1 (± 0.5)  2.0 (1.8, 2.2) | a | 2.0 (± 0.6) | a | n.s. |
|  | 40 | 1.8 (1.5, 2.2) | a | 1.9 (± 0.6)  1.9 (1.5, 2.3) | a | n.s. |
|  | Cfb^∆^ | 7.6 (± 24.5) |  | 8.7 (± 19.5) |  | n.s. |
| Monocytes  (Gpt/l) | 0 | 0.5 (± 0.1)  0.5 (0.4, 0.6) | a | 0.5 (± 0.2)  0.5 (0.4, 0.6) | a | n.s. |
|  | 10 | 0.5 (0.4, 0.6) | a | 0.4 (0.3, 0.6) | a | n.s. |
|  | 20 | 0.6 (± 0.1)  0.5 (0.4, 0.6) | a | 0.5 (± 0.1)  0.4 (0.3, 0.6) | a | 0.046 |
|  | 40 | 0.5 (0.4, 0.6) | a | 0.4 (0.4, 0.5) | a | n.s. |
|  | Cfb^∆^ | 8.0 (± 20.6) |  | 0.6 (± 25.9) |  | n.s. |
| Leucocytes  (Gpt/l) | 0 | 6.0 (± 1.3)  5.9 (5.3, 6.9) | a | 6.6 (± 1.8) | a | n.s. |
|  | 10 | 6.0 (5.3, 6.8) | a | 5.9 (± 1.6)  6.0 (5.2, 6.6) | a | n.s. |
|  | 20 | 6.2 (± 1.2)  5.8 (5.6, 6.8) | a | 6.1 (± 1.6) | a | n.s. |
|  | 40 | 6.0 (± 1.3)  5.7 (5.0, 6.7) | a | 6.1 (± 1.9) | a | n.s. |
|  | Cfb^∆^ | 3.8 (± 16.9) |  | -4.3 (-16.4, 9.5) |  | n.s. |
| Thrombocytes  (Gpt/l) | 0 | 224.6 (± 39.8) | a | 229.9 (± 33.2) | a | n.s. |
|  | 10 | 236.3 (± 39.7) | a,b | 219.8 (± 38.0) | a | n.s. |
|  | 20 | 249.1 (± 36.71) | c | 249.0 (± 41.4) | b | n.s. |
|  | 40 | 257.14 (± 58.43) | b,c | 251.4 (± 42.2) | b | n.s. |
|  | Cfb^∆^ | 6.85 (1.78, 17.14) |  | 7.0 (0.9, 11.2) |  | n.s. |
| Erythrocytes  (Tpt/l) | 0 | 4.9 (± 0.3) | a | 4.9 (± 0.4) | a | n.s. |
|  | 10 | 4.7 (± 0.2) | b | 4.7 (± 0.3) | b | n.s. |
|  | 20 | 4.8 (± 0.3) | a,b | 4.8 (± 0.3) | b | n.s. |
|  | 40 | 4.8 (± 0.4) | a,b | 5.0 (± 0.3) | a | n.s. |
|  | Cfb^∆^ | -2.3 (± 5.9) |  | -3.4 (± 4.5) |  | n.s. |
| Hemoglobin  (mmol/l) | 0 | 9.1 (± 0.6) | a | 9.1 (± 0.7) | a | n.s. |
|  | 10 | 8.7 (± 0.4) | b | 8.7 (± 0.6) | b | n.s. |
|  | 20 | 8.7 (± 0.6) | b | 8.6 (± 0.4) | b | n.s. |
|  | 40 | 8.6 (± 0.7) | b | 8.9 (± 0.5) | a | n.s. |
|  | Cfb^∆^ | -4.5 (± 6.6) |  | -4.8 (± 5.0) |  | n.s. |
| Hematocrit  (%) | 0 | 0.44 (± 0.02)  0.44 (0.43, 0.45) | a | 0.44 (± 0.03) | a | n.s. |
|  | 10 | 0.42 (0.41, 0.43) | b | 0.42 (± 0.03)  0.42 (0.4, 0.43) | b | n.s. |
|  | 20 | 0.43 (± 0.02)  0.44 (0.42, 0.45) | a | 0.43 (± 0.02) | c | n.s. |
|  | 40 | 0.43 (± 0.03)  0.43 (0.42, 0.45) | a,b | 0.44 (± 0.02) | a | n.s. |
|  | Cfb^∆^ | -2.18 (± 5.85) |  | -2.46 (± 5.14) |  | n.s. |

| RDW  (%) | 0 | 13.3 (± 0.6)  13.3 (12.9, 13.7) | a,b | 13.3 (± 0.7) | a | n.s. |
| --- | --- | --- | --- | --- | --- | --- |
|  | 10 | 13.4 (± 0.8)  13.3 (13.0, 13.8) | a | 13.1 (± 0.6) | b | n.s. |
|  | 20 | 13.0 (± 0.6)  13.0 (12.6, 13.2) | c | 12.8 (± 0.6) | c | n.s. |
|  | 40 | 13.1 (12.5, 13.6) | b,c | 12.9 (± 0.7)  13.0 (12.4, 13.3) | b,c | n.s. |
|  | Cfb^∆^ | -2.3 (± 2.2) |  | -3.6 (± 2.0) |  | 0.032 |
| MCH  (fmol) | 0 | 1.87 (± 0.08) | a | 1.84 (± 0.08) | a | n.s. |
|  | 10 | 1.87 (± 0.08) | a | 1.85 (± 0.08) | a | n.s. |
|  | 20 | 1.83 (± 0.10) | b | 1.82 (± 0.08) | b | n.s. |
|  | 40 | 1.79 (± 0.10) | c | 1.80 (± 0.07) | c | n.s. |
|  | Cfb^∆^ | -1.54 (-3.89, -0.54) |  | -1.48 (-2.75, 0.56) |  | n.s. |
| MCHC  (mmol/l) | 0 | 20.6 (± 0.5)  20.6 (20.4, 20.7) | a | 20.7 (± 0.5)  20.7 (20.4, 21.0) | a | n.s. |
|  | 10 | 20.7 (20.5, 21.0) | a | 20.8 (20.5, 21.1) | a | n.s. |
|  | 20 | 20.1 (19.8, 20.4) | b | 20.0 (19.9, 20.7) | b | n.s. |
|  | 40 | 20.1 (± 0.5)  20.1 (19.7, 20.4) | b | 20.4 (± 0.4)  20.4 (20.1, 20.6) | b | 0.038 |
|  | Cfb^∆^ | -2.4 (± 2.0) |  | -2.3 (± 2.0) |  | n.s. |
| MCV  (fl) | 0 | 90.5 (88.5, 92.8) | a,b | 88.5 (87.5, 90.0) | a | 0.031 |
|  | 10 | 90.5 (88.3, 92.8) | b,c | 89.0 (86.8, 90.0) | a | n.s. |
|  | 20 | 91.2 (± 3.7)  92.0 (88.0, 94.0) | a | 89.8 (± 4.2)  89.0 (87.0, 91.0) | a | n.s. |
|  | 40 | 89.4 (± 4.0)  89.5 (86.3, 92.0) | c | 88.3 (± 3.5)  87.5 (86.0, 89.3) | b | n.s. |
|  | Cfb^∆^ | 1.1 (-1.1, 2.2) |  | 1.1 (0.0, 2.3) |  | n.s. |

* Variables expressed as mean (± SD) and/or as median (25th, 75th percentile) depending on the statistical test that was performed; Cfb^∆^ Percentage change between baseline and the end of the intervention (week 20); Points in time without a common letter are significantly different, p < 0.05. Abbreviations: aPTT, activated partial thromboplastin time; Cfb, change from baseline; INR, international normalized ratio; MCH, mean corpuscular hemoglobin; MCHC, mean corpuscular hemoglobin concentration; MCV, mean corpuscular volume; RDW, red cell distribution width.

**Table S3:** Percentage distribution of minimum values for body weight (kg) and LDL-C (mmol/l) at the examination times (2, 4, 6, 8, 10, 12, 14, 16, 18, 20 weeks) of the MoKaRi study.

| **Week** | **Minimum Body weight (kg)** | | | | **Minimum LDL-C (mmol/l)** | | | |
| --- | --- | --- | --- | --- | --- | --- | --- | --- |
|  | **MP group** | | **MP-FO group** | | **MP group** | | **MP-FO group** | |
|  | **n** | **%** | **n** | **%** | **n** | **%** | **n** | **%** |
| 2 | 2 | 7.7 | 0 | 0.0 | 0 | 0.0 | 1 | 4.0 |
| 4 | 0 | 0.0 | 0 | 0.0 | 0 | 0.0 | 2 | 8.0 |
| 6 | 0 | 0.0 | 1 | 4.0 | 1 | 3.8 | 2 | 8.0 |
| 8 | 0 | 0.0 | 2 | 8.0 | 3 | 11.5 | 1 | 4.0 |
| 10 | 3 | 11.5 | 1 | 4.0 | 4 | 15.4 | 2 | 8.0 |
| 12 | 0 | 0.0 | 1 | 4.0 | 2 | 7.7 | 3 | 12.0 |
| 14 | 1 | 3.8 | 3 | 12.0 | 3 | 11.5 | 4 | 8.0 |
| 16 | 0 | 0.0 | 0 | 0.0 | 4 | 15.4 | 2 | 8.0 |
| 18 | 6 | 23.1 | 4 | 16.0 | 7 | 26.9 | 5 | 20.0 |
| 20 | 14 | 53.8 | 13 | 52.0 | 2 | 7.7 | 3 | 12.0 |

Abbreviations: LDL-C, low-density lipoprotein cholesterol; MP, menu plan; MP-FO, menu plan plus fish oil.

Over 50 % of participants in both groups achieved the highest weight loss after 20 study weeks. After 18 weeks, 23 % of subjects in the MP group and 16 % of subjects in the MP-FO group reached their minimum body weight. The remaining number of minimum values was distributed between weeks 2 and 14 (**Table S3**).

The lowest LDL-C concentrations were mainly seen after 8 (12 %), 10 (15 %), 14 (12 %), 16 (15 %) and 18 (27 %) weeks in the MP group. In the MP-FO group the minimum concentrations were primarily observed after 12 (12 %), 18 (20 %), and 20 (12 %) weeks (**Table S3**).

Regular analysis at two-week intervals shows that the reduction in LDL-C is observed earlier than the reduction in body weight, which confirms that weight reduction and LDL-C reduction should be considered independently of each other (**Table S3**). In particular course of the LDL values over the intervention period points out that this parameter depends strongly on the adherence with the MoKaRi concept. The test subjects stated that compliance was limited by events such as public holidays, school vacations or the start of the barbecue season. This may also be an explanation for the fact that the LDL-C was at its lowest after 18 weeks (MP: 27 %; MP-FO: 20 %)) and not after 20 weeks (MP: 8 %; MP-FO: 12 %) which most closely coincides with the start of the barbecue season in Thuringia (**Table S3**).
